# Thirty years of paediatric colonic motility assessment modalities: a systematic review

**DOI:** 10.3389/fped.2026.1951800

**Published:** 2026-09-18

**Authors:** Assia Comella, Hannah Evans-Barns, Phil Dinning, Mark Safe, Misel Trajanovska, Sebastian K. King

**Affiliations:** 1 Department of Paediatirc Surgery, The Royal Children’s Hospital, Parkville, VIC, Australia; 2 Paediatric Surgery, Murdoch Children's Research Institute, Parkville, VIC, Australia; 3 Faculty of Medicine, Dentistry and Health Sciences, The University of Melbourne, Parkville, VIC, Australia; 4 Flinders University, Bedford Park, SA, Australia; 5 Department of Gastroenterology, The Royal Children's Hospital, Parkville, VIC, Australia

**Keywords:** bowel dysmotility, colonic manometry, colonic motility, investigations, paediatric

## Abstract

**Introduction:**

Disorders of paediatric colonic motility may arise following operative repair of congenital colorectal conditions, as well as spontaneously. This may lead to symptoms of faecal incontinence, in addition to constipation. Management of such typically requires several investigations, clinic appointments and treatment strategies. Different investigation modalities are available to objectively and accurately describe gastrointestinal transit time. In the paediatric setting, most of these investigations are invasive, involve radiation, or require patient hospital admission. This systematic review summarises paediatric colonic motility investigations by compiling an assessment of modalities available over the last 31 years, their uses, advantages, and limitations.

**Methods:**

The following databases (Cochrane, EMBASE, MEDLINE) were interrogated. The keywords used were “paediatric”, “pediatric”, “colonic motility”, and “investigation”, searched as keywords and Medical Subject Heading/Emtree subject heading. Studies that were included were case reports, prospective and retrospective cohort studies, case series, and reviews discussing and comparing modalities to investigate paediatric (< 18 years old) colonic motility. Inclusion criteria included papers published in the last 31 years, including data about paediatric colonic motility investigations indications, rationale for investigation of choice, advantages and limitations. The protocol was registered with PROSPERO (ID 1034832). Study selection was conducted according to the Preferred Reporting Items for Systematic Reviews and Meta-Analyses (PRISMA) protocol.

**Results:**

The initial search yielded 1061 papers. After removing duplicates and screening abstracts and full texts, 122 papers were included for analysis. The most widely-used motility investigation types were radiopaque marker studies, scintigraphy and colonic manometry. However, these had limitations, including radiation exposure, the need for an experienced-centre, and invasiveness. Hydrocolonic ultrasonography was radiation-free, not expensive, and safely repeatable for serial monitoring or assessment, but results were operator-dependent. Newer non-invasive tests that aim to limit radiation exposure have been emerging, such as cine-MRI, wireless capsule and the electromagnetic capsule tracking system. Assessment of anorectal function was often combined with colonic motility assessment to provide a full assessment, depending upon the clinical picture and diagnostic objectives.

**Discussion:**

Over the last 31 years numerous investigations have been utilised to study bowel anatomy, function, and neuromuscular ability. Each of these modalities have advantages, but also limitations. It is crucial to know the intrinsic characteristics of each modality to help decide which is most appropriate for the clinical indication in question. Radiopaque marker studies, scintigraphy and colonic manometry were the three most widely reported colonic motility studies methods. Evidence regarding colonic motility modalities in paediatrics was limited compared with the adult population. This lack of evidence is likely due to the invasive nature of some of these investigations, costs, radiation exposure, and limited availability at specific centres. It is crucial for clinicians involved in paediatric patient care to be familiar with available tests, their properties, availabilities, and to use the most appropriate investigation to answer each specific clinical question.

**Conclusions:**

This systematic review has shown that, to date, there are several colonic motility assessment modalities that all have their advantages, limitations and different indications. It is imperative for clinicians to base their investigative plan on the specific patient, clinical/centre expertise and clinical question. In the future, there is a need to develop a non-invasive, objective, world-wide reproducible and ambulatory method that may provide information on actual day-to-day physiological colonic motility.

**Systematic Review Registration:**

https://www.crd.york.ac.uk/PROSPERO/view/CRD420251034832, PROSPERO CRD420251034832.

## Introduction

Faecal incontinence in the paediatric population may have several aetiologies. Some of these are post-surgical repair of congenital colonic conditions, such a Hirschsprung disease (HD) or anorectal malformation (ARM). About 50% of children post-surgical repair for HD experience faecal incontinence (1). Patients post-ARM repair are reported to experience defective bowel control in 20%–40% of cases (2). Faecal incontinence may also present as constipation without any prior surgical intervention. This is a common presenting complaint for paediatric patients to see their general practitioner, representing 3% of presentations (3). This percentage is even higher when focusing upon paediatric gastroenterologists visits, with 25% of presentations (4–6). As the causes of constipation vary, management strategies may be equally diverse, often requiring multiple investigations and clinic appointments. To optimise targeted therapies, evaluating colonic motility may be crucial, especially in constipated patients that fail to respond to conventional medical therapies (3).

Over the years, several investigations have been developed to study bowel anatomy, function, and neuromuscular capability. Modalities vary globally, as different countries prioritise specific tools to objectively measure gastrointestinal transit time and inform personalised therapy. These investigations are chosen based on practicality, availability, invasiveness and the clinical questions to be answered (3). While each modality offers distinct advantages, they also possess limitations. Ideally, an investigation should be specific, sensitive, and capable of guiding management, with minimal costs or side effects. However, no current investigation is perfect, and availability varies by centre. Furthermore, several well-established methods involve radiation, which must be minimised in the paediatric population (5). It is, therefore, vital to understand the characteristics of each modality to determine which is most appropriate for a given clinical indication.

The primary aim of this systematic review was to catalogue and critically appraise the diagnostic modalities, developed over the past 31 years, for assessing paediatric colonic motility. Over the years, colonic manometry in particular has become the gold standard for accurate colonic motility assessment. This tool has been constantly evolving and developing in the academic domain since the 1980s. It has then been further applied to clinical use, and in particular, in the paediatric population in the 1990s. Therefore, our systematic review explored colonic motility tools utilised since the 1990s, detailing the available assessments, focusing on their diagnostic yield, practical advantages, and current constraints (7, 8).

## Methods

Study selection was conducted according to the Preferred Reporting Items for Systematic Reviews and Meta-Analyses (PRISMA) protocol (Figure 1) and registered with PROSPERO (ID 1034832) (9). Our search encompassed Cochrane, EMBASE and MEDLINE. The searched keywords were “paediatric”, “pediatric”, “colonic motility”, “investigation”. Medical Subject Heading/Emtree subject heading searches were exploded including the following terms: “paediatric”, “pediatric”; “colonic motility”, “gastrointestinal motility”, “gastrointestinal transit”, “myoelectric complex migrating”, “peristalsis”; “investigation” (Appendix 1). Reference lists were also assessed and relevant articles included (10).

**Figure 1:**
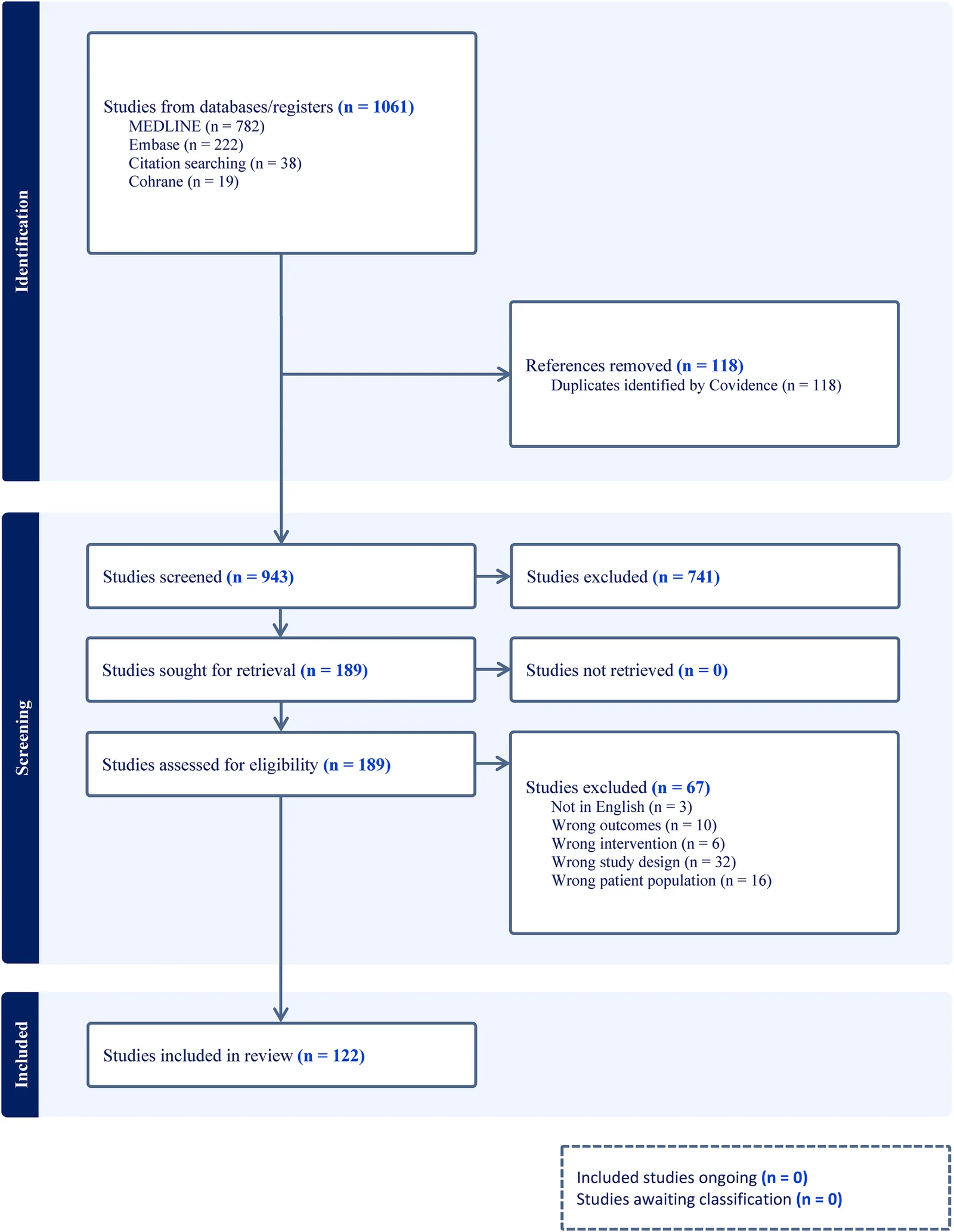
PRISMA flowchart.

Studies were uploaded to Covidence, where duplicates were removed and the remaining papers screened and analysed (11). Abstract screening and full text screening was conducted by two authors independently (AC, HEB) and screening discrepancies were discussed and agreed upon.

- Inclusion Criteria:
  - Case reports, prospective and retrospective cohort studies, case series, reviews discussing modalities to investigate paediatric (median age < 18 years old) colonic motility, that include data about paediatric colonic motility investigations indications, methodology or rationale for investigation of choice or advantages or limitations;
  - Papers studying both adult and paediatric populations were included if these data were reported separately or reporting a median age < 18 years old;
  - Papers published in the last 31 years (1995–2025), from when colonic manometry initially established its clinical diagnostic application.
- Exclusion Criteria:
  - Papers not in English;
  - Animal studies;
  - Conference abstracts or Guidelines or Letters to the Editor or Book Chapters or Opinion papers/Consensuses or Reviews;

Analysed data for each selected paper were extracted by AC. Extracted data included: author, year, country, study type, patient cohort, mean/median age or range of the cohort, colonic motility investigation modalities in children, motility investigations advantages, and motility investigations disadvantages (Appendix 2—Tables 1, 2). The risk of bias assessment was undertaken using the Modified Newcastle-Ottawa Scale (NOS) for non-randomised trials (cohort studies) (12).

## Results

The initial search resulted in 943 papers, after 118 duplicates were removed (Cochrane (13), EMBASE (222) and MEDLINE (782)). After abstract and full text screening, 122 papers were included for analysis (4, 5, 10, 13–129). (Figure 1)

The following paediatric patients were part of this review:

- Bowel dysfunction: 262
- Chronic idiopathic constipation: 2429
- Faecal incontinence: 95
- Pseudo-obstruction: 26
- Recurrent abdominal pain: 273
- Urinary tract symptoms: 20
- Children with cystic fibrosis: 22
- Severe brain damage and chronic constipation: 58
- Myelomeningocele: 51
- Spinal dysmorphism: 60
- Neurofibromatosis Type 1: 20
- Intestinal neuronal malformation: 106
- ARM: 269
- Hirschsprung Disease: 644
- Post-colon resection: 24
- Healthy cohort: 194
- Unspecified: 4003

Tables 1, 2 provide a summary of colonic motility investigations:

- The most widely-used motility investigation type was radiopaque marker studies (ROM). This transit test assesses movement of ingested radiopaque markers over days through repeated abdominal radiographic assessment. It is a useful clinical test that is not expensive and widely available. Limitations include patient compliance with attendance at appointments, accurate anatomical assessment of markers location on a plain radiograph, variable radiation exposure, and variation in protocols. The percentage of articles reporting about this investigation in this review is 35% (17–20, 24, 30–32, 34, 46, 49, 50, 55, 57, 59, 63, 73, 75–77, 79–81, 84, 86, 91, 93–95, 97, 101, 103–107, 109, 113, 116–119, 122, 125, 126).
- Scintigraphy is a non-invasive sensitive nuclear medicine imaging technique test. It involves ingestion of a radioactive tracer (Idium-111 or Technetium-99) and a variable number of acquired images using an abdominal gamma camera. It provides detailed gastrointestinal tract (stomach to rectum) and segmental colonic transit information, which may be useful for clinical management. This is a relatively expensive, specialised test that carries a radiation burden for the patients. The percentage of articles reporting about this investigation in this review is 16% (5, 21, 23, 26, 27, 46, 50, 53, 55, 75, 83, 92, 99, 100, 102, 120, 121, 128, 129).
- Colonic manometry (CM) is the current gold-standard motility assessment that is typically performed following colonoscopic placement of the manometry catheter (water-perfused, solid state, fibre optic) after bowel preparation has been undertaken. The catheter is then used to collect real-time data of colonic contractility in a number of states—following emergence from anaesthesia, prior to ingestion of a standardised meal, following ingestion of a standardised meal, and following instillation of a stimulant laxative. The test may be used to assess neuromuscular integrity and, therefore, characterise patients as having a functional or neuromuscular cause for constipation. The acquisition of these detailed data needs to be weighed against the limitations of the investigation, including the need for a general anaesthetic to place the catheter, the inpatient admission to hospital, and the specialised equipment/staff required to perform the investigation and analyse the acquired data. The percentage of articles reporting about this investigation in this review is 43% (4, 5, 10, 15, 16, 20, 22, 25, 28, 29, 33–40, 42–45, 48, 51, 52, 54–56, 58, 60–70, 72, 74, 75, 78, 82, 85, 87, 88, 90, 91, 93, 96, 98, 101, 105, 108, 110–112, 115, 124, 127).
- Ultrasonography (US) is a motility assessment tool that involves saline filling of the colon during transabdominal US assessment, providing real-time details about bowel anatomy and morphology that may affect patient's motility (ie. faecal impaction, colonic diameter). The test is radiation-free, less expensive compared with scintigraphy or colonic manometry, and provides information about volume and width of bowel loops, evaluating volume of fluid movement to clean the colon and haustra movement to assess colonic peristalsis. It is, though, limited in function-evaluation as it provides a small window of activity, with the risk of a too-short spatial and timeframe assessment to assess motility in order to capture High Amplitude Propagating Contractions (HAPC). The test is safely repeatable for serial monitoring/assessment, but it is operator-dependent with variable accuracy depending on the indications and study population. The percentage of articles reporting about this investigation in this review is 5% (14, 19, 47, 57, 71, 74, 77).
- Magnetic Resonance Imaging (MRI) has been largely utilised to provide radiation-free motility assessment and to determine colonic length. This modality has had different applications and outcomes of interest, being able to be used as a transit study as well as dynamic contractility and anatomical assessment tool. Cine-MRIs are dynamic investigations that may assess whole gut peristalsis, as well as identify areas of stasis and abnormal contractility (112, 114). Images acquired by MRI have also been used as transit assessment tools looking at movement of ingested markers capsules filled with water and a gadolinium-based contrast agent or 19F-fluorine-labelled capsules (50, 130, 131). These investigations have the added benefit of being able to define colonic length and anatomical structures/abnormalities (132–134). These investigations are expensive, require specialised skills and the patients are required to be stationary for prolonged imaging times. The percentage of articles reporting about this investigation in this review is 2%.
- There are newer, non-invasive tests that assess colonic transit that have emerged, with a specific aim of limiting radiation exposure. These include the wireless capsule endoscopy, the electromagnetic capsule tracking system, and surface electroenterography. The first two investigations involve swallowing capsules that record data as they pass through the gastrointestinal tract, with the ability to assess the whole gut transit through cameras, pH, temperature, and pressure measurements. While primarily used for research in paediatric populations, these tests have the potential to provide important information regarding colonic motility obtained while the patient is mobile and in their natural environment. Limitations include manufacturing availability, cost, availability, risk of capsule retention, and/or an inability to swallow the capsules (13, 41, 50, 86). Surface electroenterography instead involves assessment of colonic frequencies via ultrasound-guided placement of surface electrodes on the abdomen covering all parts of the colon in order to evaluate colonic motility. This is a non-invasive method, but it is still a research tool in particular in the paediatric population, which has unique challenges such as movement artefact and body size. The percentage of articles reporting about these investigations in this review is 5% (89).
- **Contrast enemas** involve the instillation of radio-opaque contrast per rectum or per stoma, with real time fluoroscopic images acquisition. Whilst colonic motility information is limited, it may be used more widely for its therapeutic effect on colon emptying and evaluation of the degree of colonic distension. The percentage of articles reporting about this investigation in this review is 6% (21, 31, 33, 84, 85, 118, 119).
- Assessment of anorectal function is often combined with colonic motility assessment to provide a full assessment depending on the clinical picture and tests indication. In clinical practice, this may be achieved with **anorectal manometry** and/or **defecography**, while **Barostat** has also been used in the academic field. The percentage of articles reporting about these investigations in this review is 25% (18, 21, 23, 33, 48, 49, 51, 56, 57, 62–64, 68, 69, 73, 75, 79, 85, 91, 93, 97, 102, 105–108, 116, 118, 119, 122).

**Table 1:** Summary table of available colonic transit investigations used in children.

Investigation	Proportion (percentage) of articles	Advantages	Disadvantages
Radiopaque Markers (17–20, 24, 30–32, 34, 46, 49, 50, 55, 57, 63, 73, 75–77, 79–81, 84, 86, 91, 93–95, 97, 101, 103, 104, 106, 107, 109, 113, 116–119, 122, 125, 126)	35%	Simple Validated Reproducible Easily accessible and widely used Non-invasive Not expensive Possible to perform in children as young as 2 years old with possibility to open the capsule to swallow the markers, well-tolerated Office-based investigation Non-absorbable markers with similar specific gravity to the GIT contents Clear identification of patients with slow transit time Availability of normal standards for paediatric population Localisation of the delay in colonic transit and useful if unreliable bowel history Differentiation between constipation and functional non-retentive faecal soiling	Radiation exposure Need to be able to swallow markers Risk of reduced compliance Variation in protocols (variation in amount of radiation exposure and hospital visits) Variation in normal values Risk of unreliable data if non-compliant patient Poorer definition of anatomical location of markers, 2D projections Timing of markers ingestion and Xray may interfere with transit time estimation Potential for observer error Poor information about segmental colonic transit No information about motility in other parts of the bowel other than the colon No information regarding the differentiation between dyssynergia or other causes of rectal outlet obstruction Risk of plastic markers following a different motion dynamic than faeces
Scintigraphy (5, 21, 23, 26, 27, 46, 50, 53, 55, 74, 75, 83, 92, 99, 100, 102, 120, 121, 128, 129)	16%	Non-invasive Safe Validated Estimation of gastric and small bowel transit Qualitative and quantitative motility assessment Possible to be performed at any age Outpatient study Accurate assessment of whole gastrointestinal and segmental colonic study Identification of whole colonic delayed transit, localised colonic dysmotility, functional rectal obstruction Identification of slow-transit constipation and functional rectosigmoid obstruction Easier colonic outlining, with provision of more details and greater sensitivity than solid markers studies Standard radiation exposure (equivalent to two abdominal radiographs) Low interobserver variability More physiological evaluation than measuring motility administering solid ingestible pellets, with availability of assessment of multiple images Useful in predicting patients that are likely to have abnormalities in colonic manometry Localisation of the delay in colonic transit and useful if unreliable bowel history; Differentiation between constipation and functional non-retentive faecal soiling	Radiation exposure Expensive Swallowing of capsule containing radiopharmaceuticals Relatively new method with requirement for specialised staff and equipment Short window of timing for imaging due to radiation decay Need for several scans with assessment over several days Repeated hospital visits Risk of low compliance Overlapping bowel segments may reduce accuracy Variation in identified number of regions with lack of standardised protocol Lack of age-specific normal values for regional transit measures Faecal impaction at time of the study may affect results Difficult to perform and requires selective intubation of the digestive tract Wide range of normal values Variability in protocol and reporting methods with lack of standardisation Use of radioactive materials causing ionising radiation effects Assessment of bulk transit only and no detailed assessment of motor patterns
Colonic Manometry (4, 5, 10, 15, 16, 20, 22, 25, 28, 29, 33–40, 42–45, 48, 51, 52, 54–56, 58–70, 72, 74, 75, 78, 82, 85, 88, 90, 91, 93, 96, 98, 101, 105, 108, 110–112, 115, 124, 127)	43%	Discrimination between functional and neuromuscular causes of constipation Provision of objective evidence of colonic motility Full evaluation of colonic motor function with information about whole colonic and segmental colonic transit Direct assessment of muscle contraction and neuromuscular integrity High-resolution manometry may further identify colonic pathophysiology detecting more propagating contractions Assessment for underlying neuropathy or myopathy or behavioural causes for constipation Guidance for further therapeutic intervention Quantitative and qualitative motility assessment, with visually-intuitive “spatiotemporal plot” of colonic pressure activity Solid state manometry allows patients to be ambulatory during the test allowing for longer data recording and closed to physiological state Clear indications and standardised interpretation More paediatric data available with water-perfused catheters Useful investigation in predicting response to surgical interventions and the eventual need for surgery	Invasive Expensive General anaesthetic requirement Hospital admission requirement Bowel preparation and gas insufflation requirement, which may affect results Risk of bowel perforation during catheter placement No assessment of movement of faeces nor differentiation between fast and slow transit Lack of motility data in healthy children Study-interpretation expertise required Variation in protocols Limited availability, being only performed at specialised centres with specialised staff and equipment (ie. availability of endoscopes and catheters sizes for small children) Risk of abdominal cramps, frequent bowel motions in setting of stimulant medications that is being given Length of the investigation, also having to lay in bed during study Lack of normal values in paediatrics Risk of water overload using eater-perfused catheters Colonic manometry not predictive of medical changes in management
Ultrasonography (14, 19, 47, 57, 71, 77)	5%	Usefulness in displaying the width of bowel loops and evaluating colonic peristalsis Lack of radiation exposure Limited invasiveness Reduced costs Available control paediatric population data Repeated evaluation at different time points making useful as follow-up tool Determination of stool texture, activity of the colon and appropriate volume needed to fill up and clean the colon	Variable accuracy depending on the indications and study population Inter-user variability
Breath tests (46, 94, 99)	2%	Non-invasive Simple Well tolerated by children Able to detect sugar malabsorption	Orocaecal transit time, which is of little importance in the physiopathology of constipation
Cine-MRI (112, 114)	2%	Excellent soft tissue contrast with information about pelvic anatomy, colonic volume, colonic wall movement, freely mobile water content, chyme relaxometry, gas consent and movement of the pelvic floor during defecation Spatio-temporal mapping technique Information about segmental motility of the colon Possible to avoid general anaesthesia to perform the test Assessment for lumbosacral spine abnormalities Lack of radiation Non-invasive Increasingly available Fast acquisition of information Large number of parameters of gastrointestinal motor function obtained from a single study High resolution and multiplanar capability	Mainly research-based modality Need for specific software and staff Difficult data interpretation for controls Need for general anaesthetic in the paediatric population therefore obviating the ability to image voluntary defecation No evidence to be used if no suspicion for neurologic abnormalities Not possible to be undertaken if metal implants present Expensive Need for further validation and harmonisation of protocols Subjective analysis Need for further validation in paediatrics Lack of paediatric-specific normative data No data showing comparability with colonic manometry in paediatrics
Wireless motility capsule (41, 50, 86)	2%	Non-radioactive and non-invasive Measurements of gastrointestinal transit and motor function, intraluminal pH, pressure and temperature Assessment of the whole bowel's function Potential to offer a standardised ambulatory approach Ease of us and tolerability by patients	Recording over a 5-day period transmitting data about pH, temperature, pressure Lack of data/use in children Fasting prior to capsule ingestion Unable to characterise propagating contractions No assessment of segmental colonic transit time nor quantification of colonic mass movements Practical difficulty of children swallowing the capsule, as well as capsule retention risk Need for specialised equipment and staff Single sensor not allowing for characterisation of propagating contractions Limited role in clinical evaluation and management at this stage
Electromagnetic capsule tracking system (13, 50)	2%	Ambulatory Radiation-free Minimally-invasive Precise assessment of progression and simultaneous tracking of more than one capsule Detailed information about colonic motility patterns Measurements of gastric, colonic and total transit time Measurement of colonic transit and length High spatial and temporal resolution with continuous monitoring of transit	No available paediatric data Potential difficulty swallowing capsule and risk of capsule retention Standardised side of capsules despite different ages Restriction in some daily activities during the investigation Manual analysis Non-direct measurement of gastrointestinal contractions
Surface electroenterography (89)	1%	Non-invasive Assessment of segmental colonic function Measurement of gastrocolic reflex Potential for ambulatory monitoring	Non-validated in children Artefacts Paediatric challenges (body size)
Contrast enema (21, 31, 33, 84, 85, 118, 119)	6%	Therapeutic effect of emptying the colon Evaluation of degree of colonic distension Assessment for anatomical strictures and colonic anatomy Useful for longitudinal follow-up	Radiation exposure Invasive Anatomical only information, no functional information: not able to assess severity of constipation
Anorectal manometry (18, 21, 23, 33, 46, 48, 49, 51, 55–57, 62–64, 68, 69, 73, 75, 79, 85, 93, 102, 105–108, 116, 122)	20%	Information regarding anal sphincter pressure, sensory function, recto-anal inhibitory reflex (RAIR) and defecation dynamics Assessment of sensorimotor activity of the rectum and anal region Possible to perform at any age (though with data interpretation limitation for patients aged less than 1 year old) 3D dynamic representation of pressure changes within the anal canal both radially and longitudinally	Lack of standardisation in methodology Artefacts Requirement for anaesthesia/sedation Lack of data about normal anorectal manometry pressures in children Need for patient cooperation for sensory testing and dynamic components of the test Children <5yo and patients under general anaesthetic have a limited assessment Small risk of colonic perforation Need for an empty rectum Lack of validated and reproducible normative values for children
Defecography: (64, 75, 118, 119, 144) Fluoroscopic Magnetic resonance	4%	Evaluation of both morphological and dynamic factors of defecation More physiological positioning of the patient while imaging is taken, impacting less on pelvic floor physiology and dynamics of defaecation Multiplanar capability Visualisation of pelvic organs	Clinical significance is debatable Lack of paediatric studies validating these methods
Barostat (133)	1%	Assessment of rectal tone and compliance	Psychological bias in response to stimuli Current use is limited to research

**Table 2:** Comprehensive table summarising each modality according to most common identified disadvantages.

Investigation modality	Radiation exposure	Invasiveness	Inpatient study	Multiday study	Need for specialised equipment/staff	Increased cost	Lack of information about segmental colonic motility	Lack of standardised protocol/reporting system	Limited paediatric evidence
Radiopaque Markers (17–20, 24, 30–32, 34, 46, 49, 50, 55, 57, 63, 73, 75–77, 79–81, 84, 86, 91, 93–95, 97, 101, 103, 104, 106, 107, 109, 113, 116–119, 122, 125, 126)	x			x			x	x	
Scintigraphy (5, 21, 23, 26, 27, 46, 50, 53, 55, 74, 75, 83, 92, 99, 100, 102, 120, 121, 128, 129)	x			x	x	x		x	
Colonic manometry (4, 5, 10, 15, 16, 20, 22, 25, 28, 29, 33–40, 42–45, 48, 51, 52, 54–56, 58–70, 72, 74, 75, 78, 82, 85, 88, 90, 91, 93, 96, 98, 101, 105, 108, 110–112, 115, 124, 127)	x	x	x		x	x		x	x (lack of paediatric reference values)
Ultrasonography (14, 19, 47, 57, 71, 77)							x	x	
Breath test (46, 94, 99)							x		x
Cine-MRI (112, 114)			x (if general anaesthetic required)		x	x			x
Wireless motility capsule (41, 50, 86)				x	x	x			x
Electromagnetic Capsule Tracking (13, 50)				x	x	x			x
Surface electroenterography (89)					x				x
Contrast enema (21, 31, 33, 84, 85, 118, 119)	x	x					x		
Anorectal manometry (18, 21, 23, 33, 46, 48, 49, 51, 55–57, 62–64, 68, 69, 73, 75, 79, 85, 93, 102, 105–108, 116, 122)		x	x						x (lack of paediatric reference values)
Defecography: (64, 75, 118, 119, 144) a. Fluoroscopic b. Magnetic resonance	x	x							x (lack of paediatric studies validating this)
Barostat (133)		x					x		x

Risk of bias assessment was undertaken using the Modified Newcastle-Ottawa Scale (NOS) for non-randomised trials (Appendix 3—Table 5) (12). The results revealed 73% of the articles fulfilled three or more of the four subgroups in the Selection-domain; though 75% did not have a comparator cohort. Furthermore, 47% fulfilled three of the Outcome-domain subgroups with the majority not having long-term follow-up. The overall average score was 6.

## Discussion

This review explored advantages and limitations of various motility investigations used in the last 31 years. Radiopaque marker studies, scintigraphy and colonic manometry are the three most widely used colonic motility investigations (3). Radiopaque marker studies involve ingestion over a day or consecutive days of one or more capsules. Abdominal radiographs are then performed, usually on day 3 and/or day 7 post-capsule ingestion, to assess the colonic course of the makers. In Australia an abdominal radiograph costs AUD$54.70 with a package of sitz markers averaging a cost of AUD$200–1,500, while scintigraphy has a cost of AUD$728.85 (135–140). Historically, radiopaque markers testing is a widely-used investigation that has paediatric evidence supporting its validity (132, 141). This investigation though exposes children to radiation, is at risk of observer error and has poor definition of segmental colonic transit as a limited number of serial x-rays are taken (3, 46). Rybak et al. describe this investigation as an initial platform to ascertain which children might require further, potentially more invasive, assessment (142).

Scintigraphy provides useful information about whole gastrointestinal and segmental colonic transit, as well as gastric and small intestine function, while still being non-invasive as it involves swallowing of a radioactive capsule and monitoring of its passage throughout the whole intestine. Carmo et al. (21), through scintigraphy, showed that different portions of the colon have different functions, with the transverse colon working as reservoir and the descending colon as conduit. However, scintigraphy also exposes children to radiation, is expensive, is only provided at specialised centres for the paediatric population, and lacks age-specific normal values (140, 143). Limitations for radiopaque markers studies and scintigraphy studies also involve variation in protocols, which makes comparison of data difficult across different centres. For example, Popescu et al. presented a clear summary of the different ROM protocols that are available, and Rajundrajith et al. discussed variation in protocols and investigation reporting (3, 137, 144). Low compliance with the test and risk of loss-to-follow up are present with both radiopaque studies and scintigraphy. Papadopulos et al. (137) reported the ESPGHAN/NASPGHAN 2011 evidence-based recommendations, which discouraged the use of scintigraphy as a routine investigation for children with functional constipation.

Colonic manometry in a paediatric setting involves a general anaesthetic and hospital admission for placement of a catheter per rectum or per stoma in the colon. The catheter may be water-perfused or a solid-catheter (142). Since the early 2000s the advance of high-resolution colonic manometry, with sensors placed at only 1–2 cm distance from each other, has become the gold-standard for assessing colonic neuromuscular function in children (8, 145, 146). Colonic manometry provides evaluation of segmental colonic motor function, potentially discriminating between functional and neuromuscular causes of constipation. This is a very useful investigation, but it has some limitations: it is invasive and expensive and therefore of limited availability, requiring a general anaesthetic and hospital admission, as well as specialised equipment and staff. Acquisition of the equipment and software to perform high resolution colonic manometry may range from AUD$20,000 to AUD$100,000 (147). Bowel preparation is required prior to the test, raising the question if the data obtained are truly representative of the daily physiological state of the colon.

Most importantly, there is a lack of paediatric control data with colonic manometry as a healthy paediatric cohort has never been investigated due to the invasiveness of the test and the unethical nature of the potential study. Tipnis et al. and Dranove et al. compared the use of ROM and CM in the paediatric population. They concluded that that ROM has a role as a screening test to rule out colonic inertia though with limitations regarding segmental information. CM can then be used and become useful in further describing to drive specific management potentially distinguishing between functional and neuromuscular disorders (34, 101). Additionally, Rybak et al. agreed with Papadopulos et al. that, although there is accordance between scintigraphy and colonic manometry regarding constipation categorisation, scintigraphy suggestive of colonic inertia should undertake further assessment (with colonic manometry) prior to proceeding with medical or surgical management, as scintigraphy provides information about bulk transit without detailed assessment of motor patterns which may be crucial for targeted treatment (137, 142). Another limitation of colonic manometry is the lack of agreement on definition of “normal colonic motility”. American and European Consensuses have been drawn in the past to attempt to describe this, but there is still lack of standardised data reporting. This makes international comparison of findings challenging for an investigation that already has limitations in terms of number of centres being able to perform it and interpret it (87, 142, 148–150). Over the last 30 years, high-resolution colonic manometry (HRCM) has revolutionised the understanding of colonic motility, being able to directly assess bowel contractility and providing insight in colonic myogenic and neurogenic function (145, 151, 152). With sensors being placed just a few cm from each other, it has finally been possible to describe the complex interactions of different contractions (propagating vs. non propagating, anterograde vs. retrograde). This has then become the gold-standard investigation to assess colonic motility.

Over the years, attempts have been made to develop more specific and less invasive modalities to describe colonic motility. These have mainly been studied in the adult population, with only minimal data available in the paediatric population and predominantly in the research field. Cine-MRI is a non-invasive, radiation-free, dynamic high-resolution spatiotemporal investigation that allows visualisation of bowel diameter as well as information about colonic motility (153). However, this is an expensive modality that has limited clinical use and applicability currently as its data yield is still emerging and being defined with further studies needed to prove its clinical validity (142, 144, 148, 149, 154). Another assessment modality was the Wireless Capsule (SmartPill Corporation, Buffalo, NY), which was discontinued in 2023 due to ongoing challenges accessing its manufacturing components (41, 142, 155). Haase et al. first described the 3D-Transit system, which was compared with ROM and found to have better estimation of segmental transit time and provide details about colonic motility patterns (50, 156, 157). The 3D electromagnetic Capsule Tracking system is an ambulatory radiation-free system and has the ability to precisely assess the progression and tracking of multiple swallowed capsules, providing information about the whole gastrointestinal motility transit time with high spatial and temporal resolution (13). On the other hand, this device has mainly been used in the academic field, with limited paediatric data available (13, 50). In addition, the capsules have a standardised size with the potential to be difficult to swallow or to be retained in children (142). Lastly, surface electroenterography is a new emerging non-invasive tool that assesses colonic motility via US-guided placement of electrodes on the abdomen. This has mainly been used in the academic area and is yet to be validated in the paediatric population (89). All these emerging newer investigations strive to be non-invasive, artefact free and limit patient distress, while accurately assessing colonic physiology. However, there are limitations to their clinical use. They are costly and require specialised equipment and trained staff. Moreover, the large amount of data generated from these new investigations also poses a challenge in terms of length of data analysis. Therefore, the use of artificial intelligence (AI) to support analysis in this space is fast expanding (158).

In conjunction with colonic motility, anorectal function assessment is also crucial in patients that present with refractory constipation, as rectal distension may cause inhibition of colonic motor activity (5, 159, 160). Indeed the rectosigmoid function is crucial for stool evacuation, with this anatomical area having been identified as the physiological gatekeeper for stool evacuation through a physiological feedback regulatory mechanism (161). Anorectal manometry is a useful test to acquire information about anal sphincter pressure, sensory function, recto-anal inhibitory reflex (RAIR) and rectum-external sphincter defecation dynamics. If a 3D catheter is used, 3D dynamic representation of pressure changes in the anal canal may also be graphically represented. This modality often requires general anaesthesia in the younger paediatric population due to its invasive nature. As a result, only limited information may be gained as patient cooperation is needed for sensory testing and some dynamic components of the test. Defecography may provide information about morphological and dynamic defecation whilst the patient is in a physiological position. Similarly, contrast enema may provide evaluation of colonic morphology and dimensions, as well as having a therapeutic effect to empty the colon, but this provides little information about colonic motility.

This systematic review has limitations. Several of the articles lacked comparability as they are mainly case series or single arm cohort studies with small cohort of studied patients. Some of the articles also lacked details about length of patient follow-up and a comparator cohort, but most of them had clear and structured population selection and assessment of outcome, as highlighted by the Modified Newcastle-Ottawa scale assessment results. This systematic review only includes three randomised controlled trials, with a range of retrospective or prospective studies, case series or cohort or case control studies Due to the heterogeneity of included studies, patient populations, diagnostic protocols and outcome measures, only a qualitative narrative analysis could be performed with no meta-analysis. This reflects the lack of standardised protocols for these investigations, which poses a challenge to comparing international data and drawing conclusions to optimise patient management. Despite these limitations, this systematic review includes a wide range of articles, including 122 publications from multiple countries and centres across the world.

In summary, evidence regarding colonic motility modalities in paediatrics is scattered compared with the adult population. This lack of evidence is likely due to the invasive nature of some of these investigations, as well as costs, radiation exposure and limited availability at specific centres. Comparison and reproducibility of existing data is also complicated due to high variability in protocols and reporting. Based on the evidence, the authors conclude that site investigation availability and staff expertise pose the first limitations to the clinical use of each of these. Colonic manometry is currently the gold-standard to assess colonic motility. The emerging use of surface mapping provides a newer non-invasive alternative that can use electrical frequencies as surrogate markers to measure colonic motility. This has the potential to become an alternative to colonic manometry. If the clinical question requires transit information, scintigraphy provides important whole gut and segmental information. This review highlights that it is crucial for clinicians involved in patient care to be familiar with available tests, their properties, availabilities, and to use these investigations in conjunction with each other. This will ensure they have all relevant information to answer the clinical question and provide a targeted care plan for the specific patient

## Conclusions

This systematic review showed that to date there are several colonic motility assessment modalities that all have their advantages, limitations and different indications. It is imperative for clinicians to base their investigative plan on the specific patient, clinical/centre expertise and clinical question. Clinicians should make patients and their families aware that this can be a lengthy process that might require a combination of different investigations in order to have the most optimal answer and individualised management. In the future, there is a need to develop a non-invasive, objective, world-wide reproducible and ambulatory method that may provide information on actual day-to-day physiological colonic motility. If such a modality was achieved, this could allow measurement in control healthy cohorts to produce normative data for comparison and optimally target differences.

## Data Availability

The original contributions presented in the study are included in the article/Supplementary Material, further inquiries can be directed to the corresponding author.
