# Supplementary material for: Thirty years of paediatric colonic motility assessment modalities: a systematic review
Source: Front Pediatr. 2026 Sep 18;14:1951800. doi: 10.3389/fped.2026.1951800 (PMC13630753; doi:10.3389/fped.2026.1951800)
Supplement: Supplementary file 2 [file Supplementaryfile2.docx]

## **Appendix 2 - Table 1:** Summary demographic information of each paper.

| **Author** | **Year** | **Country** | **Study Type** | **Patient cohort undergoing motility study** | **Mean/median age or range of the cohort** |
| --- | --- | --- | --- | --- | --- |
| Staiano A. *et al.*(97) | 1996 | Italy | Prospective randomised double-blind placebo-controlled trial | 20 children with severe brain damage and chronic constipation:  Cisapride group: 10 children  Placebo group: 10 children | Cisapride group: 6.4 +/- 4.6 years  Placebo group: 5.1 +/- 4 years |
| Pigeon N. *et al.*(79) | 1997 | Canada, France | Prospective case-control study | Intervention cohort: 22 children with myelomeningocele  Control cohort: 22 healthy children | Mean: 11.5 years  Range: 7-21 years |
| Rintala RJ. *et al.*(84) | 1997 | Finland | Prospective cohort study | Intervention group: 90 children with anorectal malformations  Control group: 25 healthy children | Intervention group:  Median: 7 years  Range: 3-13 years  Control group:  Median: 8 years  Range: 3-14 years |
| Hamid SA. *et al.*(124) | 1998 | USA | Prospective cohort study | 40 children undergoing colonic manometry:  26 with chronic intestinal pseudo-obstruction (CIP)  9 with functional faecal retention (FFR)  5 with unspecified functional bowel disorder with constipation (FBD) | Mean: 6.5 years  Range: 17 months - 17 years |
| Shankar KR. *et al.*(106) | 1998 | Turkey | Retrospective cohort study | 40 children with faecal incontinence  14 with spinal dysmorphism  26 other | Median: 11 years  Range: 6-21 years |
| Baillie CT. *et al.*(15) | 1999 | UK | Prospective cohort study | 64 children with Hirschsprung Disease who underwent Duhamel procedure | Range: birth-16 years |
| Chung KS. *et al.*(23) | 1999 | South Korea | Prospective cohort study | 120 children with recurrent abdominal pain | Mean: 9.6+/-2.6 years  Range 4.8-14.5 years |
| Halabi IM *et al.*(123) | 1999 | Jordan | Prospective, randomised, double-blind, placebo-controlled clinical trial | 64 children with chronic idiopathic constipation | Mean:  cisapride group: 8.45 +/- 2.42 years  control group: 8.26 +/- 2.43 years |
| Heikenen JB. *et al.*(51) | 1999 | USA | Prospective cohort study | 13 children with ARM | Mean: 8.6 years  Range: 5-13 years |
| Ure BM. *et al.*(104) | 1999 | Switzerland | Prospective cohort study | 106 children with intestinal neuronal malformations | Mean: 4.9 +/- 5.6 years |
| Wheatley JM. *et al.*(128) | 1999 | Australia | Prospective cohort study | 100 children with constipation refractory to medical treatment | Mean: 7.9 years  Range: 7 months – 19 years |
| Di Lorenzo C. *et al.*(32) | 2000 | USA | Prospective cohort study | 46 children after surgical management of Hirschsprung Disease | Mean: 5.5+/-3.3 years  Range: 16 months-14 years |
| Lucanto C. *et al.*(68) | 2000 | USA | Retrospective case series | 11 children | Mean: 8.1 years  Range: 3-18 years |
| Miele E. *et al.*(73) | 2000 | Italy | Prospective cohort study | 21 children | Mean: 11.8+/-4 years  Range: 3.4-17 years |
| Guimaraes EV. *et al.*(49) | 2001 | Brazil | Prospective cross-sectional study | 38 children with chronic functional constipation | Mean: 7.2+/-0.3 years  Median: 7.5 years  Range: 4-14 years |
| Treepongkaruna S. *et al.*(102) | 2001 | Australia | Retrospective cohort study | 35 children with severe chronic constipation:  Substance P deficient: 25 children  Substance P normal: 10 children | Substance P deficient – mean: 8.2 +/- 0.9  Substance P normal: 9.2 +/- 0.9 years |
| Villarreal J. *et al.*(111) | 2001 | USA | Retrospective cohort study | 12 children | Mean: 4 years  Range: 2-14 years |
| Gutierrez C. *et al.*(46) | 2002 | Spain | Prospective case-control study | 68 children:  38 patients with encopresis;  30 patients with normal bowel habits; | Range: 2-14 years |
| Shin YM. *et al.*(92) | 2002 | Korea, Australia | Retrospective cohort study | 180 children with severe, treatment-resistant constipation | Slow Transit Constipation - Mean: 10.5 years  Functional Faecal Retention - Mean: 6 years |
| Pensabene L. *et al.*(78) | 2003 | USA | Retrospective cohort study | 146 children (150 colonic manometry studies) | Median: 79 months  Range: 4-225 months |
| Benninga MA. *et al.*(16) | 2004 | The Netherlands | Prospective cohort study | 215 children with defecation disorders or abdominal pain referred to the paediatric motility unit | Paediatric constipation group:  Mean: 8 years  Range: 5–14  Functional non-retentive faecal soiling group:  Mean: 9 years  Range: 5–17 years  Recurrent Abdominal Pain group:  Mean: 9 years  Range: 5–16 years |
| Chitkara DK. *et al.*(22) | 2004 | USA | Prospective cohort study | 67 children undergoing anorectal manometry  41 children undergoing scintigraphy | Mean: 14.7+/-3.3 years |
| De Lorijn F. *et al.*(29) | 2004 | The Netherlands | Prospective cohort study | 169 children referred to paediatric gastroenterology outpatient | Median: 8.4 years /7.0-10.5) |
| Langer J. *et al.*(63) | 2004 | Canada | Retrospective case series | 49 children | Median: 4 years  Range: 1-17 years |
| Martin MJ. *et al.*(70) | 2004 | USA | Prospective cohort study | 9 children with refractory functional colonic obstruction | Mean: 4.8 years  Range: 5 weeks – 17 years |
| Park ES. *et al.*(76) | 2004 | Korea | Prospective cross-sectional study | 38 children with spastic cerebral palsy | Mean: 5+/-2.9 years |
| Wagener S. *et al.*(113) | 2004 | UK | Prospective cross-sectional study | 22 healthy children | Median: 10 years  Range: 4-15 years |
| Zaslavsky C. *et al.*(117) | 2004 | Brazil | Prospective cohort study | Functional constipation group: 48 adolescents  Control group: 13 children | Mean: 12 +/- 2 years  Range: 12-18 years |
| Gertken JT. *et al.*(43) | 2005 | USA | Retrospective case series | 173 children with constipation referred for colonic manometry | Mean: 6.8 years  Range: 1-17 years |
| Soares ACF. *et al.*(94) | 2005 | Brazil | Prospective cohort study | 40 children with chronic constipation | Range: 3-12 years |
| Stanton MP. *et al.*(98) | 2005 | Australia | Prospective case-control study | 6 children with slot transit constipation | Mean: 11.5 years  Range: 8-16 years |
| Watanabe Y. *et al.*(114) | 2005 | Japan | Prospective cohort study | 26 paediatric patients | Median: 10 years  Range: 3-17 years |
| Zhang SC. *et al.*(118) | 2005 | China | Retrospective cohort study | Intervention; 22 children who underwent one-stage transanal endorectal pull-through for HD  Control: 22 healthy children | Mean: 4 years  Range: 12 months – 13 years |
| Zuccarello B. *et al.*(121) | 2006 | Italy | Retrospective cohort study | 32 children with anorectal malformations  18 children with functional constipation | Anorectal anomalies patients median age: 5.8 years  Functional constipation patients median age: 6.7 years |
| Bijos A. *et al.*(17) | 2007 | Germany | Prospective cohort study | Intervention: 120 children referred to paediatric gastroenterology outpatient for chronic constipation  Control: 105 children with normal defaecation pattern, treated for chronic abdominal pain/food allergies | Intervention:  Mean: 6.25 years  Range: 1.6-17.9 years  Control:  Mean: 8.25 years |
| Kohno M. *et al.*(60) | 2007 | Japan | Retrospective cohort study | 21 children with HD  - 8 children underwent colonic manometry | Manometry cohort:  - Median 34 months  - Range: 26-52 months |
| Zhang SC. *et al.*(119) | 2007 | China | Retrospective cohort study | 346 children post-Swenson’s pull-through | Mean: 2 years  Range: 3 months – 6 years |
| Demirogullari B. *et al.*(30) | 2008 | Turkey | Prospective cohort study | 41 children with anorectal malformation classified according to the Krickenbeck consensus | Median: 7.7 years  Range 3-25 years |
| King SK. *et al.*(58) | 2008 | Australia | Prospective case-control study | 26 children: 18 with slow transit constipation, 8 with no slow transit constipation | Mean: 12+/-0.7 years  Range: 6.6-18.7 years |
| Venkatasubramani N. *et al.*(110) | 2008 | USA | Retrospective case series | 10 children | Mean: 9.6 years  Range: 4-12 years |
| Yuan Z. *et al.*(116) | 2008 | China, Australia | Prospective case-control study | 113 children with bowel dysfunction | Children with bowel dysfunction group: Mean: 8.5 +/- 2.9 years  Control children group: Mean: 8.41 +/- 2.73 years |
| Aspirot A. *et al.*(14) | 2009 | Canada | Retrospective case series | 7 paediatric patients who underwent colonic manometry for defaecation dysfunction after 6 months of treatment | Median: 12 years  Range: 3-15 years |
| Clarke MCC. et al.(26) | 2009 | Australia | Randomised controlled trial | 26 children | Range: 7-18 years |
| Clarke MCC. *et al.*(25) | 2009 | Australia | Prospective cohort study | 7 children with slow transit constipation resistant to standard medical therapy | At time of first scintigraphy:  Mean: 7 years  Range: 5.4-10.8 years  At time of second scintigraphy:  Mean: 11.4 years  Range: 9.7’14.2 years |
| Soares ACF. *et al.*(95) | 2009 | Brazil | Prospective cohort study | 34 children with chronic functional constipation | Median: 7.8 years  Range: 3-12 years |
| Sutcliffe JR. *et al.*(100) | 2009 | Australia | Retrospective cohort study | 64 children with chronic idiopathic constipation | - |
| Dranove R. *et al.*(4) | 2010 | USA | Prospective cohort study | 20 paediatric patients referred for colonic manometry at the James Whitcomb Riley Hospital for Children | Mean age: 10.7 ± 5.4 years  Range: 2-25 years |
| Gomez R. *et al.*(45) | 2010 | USA | Prospective cohort study | 13 children undergoing colonic manometry | Mean: 9.4+/-5.8 |
| Koh HL. *et al.*(59) | 2010 | Korea | Retrospective cohort study | 96 children:  76 children with gastrointestinal symptoms  20 healthy children (control) | Mean: 10.1+/-2.67 years  Range: 5.6-15.4 years |
| Liem OB. *et al.*(67) | 2010 | The Netherlands, USA | Prospective cohort study | 23 children  14 normal motility  6 abnormal motility | Normal motility group:  Median: 11 years  Range: 7.7.20.1 years  Abnormal motility group:  Median: 9.2 years  Range: 6.2-16.3 years |
| Märzheuser S. *et al.*(71) | 2010 | Germany | Prospective cohort study | 30 children with ARM | Range: 4–17 years |
| Grasshoff-Derr S. *et al.*(47) | 2011 | Germany | Prospective cohort study | All children treated for defecation problems in the Department of Surgery  Subgroup analysis: patients with ARM and consecutive faecal incontinence (n = 63, 25 females and 38 males) | Subgroup:  Median age: 8.4 years  Range: 4-22 years |
| Kaul A. *et al.*(56) | 2011 | USA | Retrospective cohort study | 59 children who have undergone pull-through surgery for HD | Mean: 6.5+/-3.5 years  Range: 16 months – 15 years |
| vd Baan-Slootweg OH. *et al.*(105) | 2011 | The Netherlands, USA | Prospective cohort study | 91 children with morbid obesity | Median: 15 years  Range: 8-18 years |
| Clarke MCC. *et al.*(24) | 2012 | Australia | Prospective cohort study | 8 children chronic constipation and appendicostomies in situ | Range: 9-19 years |
| Liem O. *et al.*(66) | 2012 | The Netherlands, USA, Australia, India | Prospective cohort study | 15 children with constipation | Range: 5.6-16.3 |
| Rodriguez L. *et al.*(108) | 2012 | USA | Retrospective case series | 15 children | Mean: 9.9 +/- 4.5 years |
| Sood MR. *et al.*(96) | 2012 | USA | Retrospective observational reliability study | 57 colonic manometry studies in children | N/A |
| Tipnis NA. *et al.*(101) | 2012 | USA | Retrospective cohort study | 24 children with refractory constipation | Normal transit study cohort median age: 9.2 ± 4.9 years  Slow transit study cohort median age: 11.9_± 4.3 |
| Yik YI. *et al.*(129) | 2012 | Australia, Malaysia | Prospective cohort study | 32 children with treatment-resistant slow-transit constipation (STC) | Mean: 8.3 years  Range: 3–17 years |
| Firestone Baum C. *et al.*(40) | 2013 | USA | Retrospective case series | 410 children | Mean: 7.6 years  Range: 0.25-26 years |
| Giorgio V. *et al.*(44) | 2013 | UK | Prospective cohort study | 18 children with refractory slow-transit constipation (STC) and 6 control children with normal colonic transit | STC group:  Median: 7.5 years (4-15 years)  Control group:  Median: 9 years (3-10 years) |
| Hedsund C. *et al.*(50) | 2013 | Switzerland | Prospective cohort study | 21 healthy children | Median age: 10 years  Range: 7–12 years |
| Keshtgar AS. *et al.*(57) | 2013 | UK | Prospective cohort study | 92 children with chronic idiopathic constipation | Median: 8.46 years  Range: 3.35-14.97 years |
| Marte A. *et al.*(125) | 2013 | Italy | Prospective cohort study | 16 children with chronic constipation secondary to myelomeningocele | Range: 4-17 years |
| Mugie SM. *et al.*(74) | 2013 | USA | Retrospective cohort study | 26 children with severe constipation unresponsive to standard medical treatment | Mean: 11.4 years  Range: 6-19 years |
| Pedersen CE. *et al.*(77) | 2013 | Denmark | Prospective cross-sectional study | Intervention cohort: 20 children with neurofibromatosis Type 1;  Control cohort: 23 children | Intervention cohort:  Mean: 8.2 +/- 2.4 years  Range: 4-11 years  Control cohort:  Mean: 9.1 +/- 2.7 years |
| Rodriguez L. *et al.*(88) | 2013 | USA | Retrospective cohort study | 40 children with refractory constipation | Mean: 8.8 +/- 3 years  Median: 8 years  Range: 3-16 years |
| Velde SV. *et al.*(107) | 2013 | Belgium | Prospective cohort study | 40 children | Median: 11 years  Range: 6-19 years |
| Velde SV. *et al.*(109) | 2013 | Belgium | Prospective cross-sectional study | 54 children and adolescents | Median: 10 years  Range: 3-18 years |
| El-Chammas KI. *et al.*(39) | 2014 | USA | Prospective cohort study | 10 children with constipation | Median: 7.7 years (0.9-21.7 years) |
| Kerur B. *et al.*(64) | 2014 | USA | Retrospective case series study | 14 paediatric patients with chronic idiopathic constipation undergoing total abdominal colectomy with ileorectal anastomosis | Range: 10-21 years |
| Liem O. *et al.*(65) | 2014 | Australia, The Netherlands | Prospective cohort study | 38 children enrolled, 19 studies analysed | Mean: 9.4+/-0.9 years  Range: 3.9-16.3 years |
| Zivkovic VD. *et al.*(120) | 2014 | Serbia | Prospective cohort study | 101 children:  Bowel Bladder Dysfunction (BBD) group: 38 children  Constipation without urinary symptoms group: 43 children  Lower urinary tract symptoms group: 20 children | BDD group: 8.81 +/- 0.52 years  Constipation group: 6.62 +/- 0.51 years  Control group: 7.35 +/- 0.73 years |
| Carmo RLML. *et al.*(20) | 2015 | Brazil | Prospective cohort study | 28 children with functional constipation | Mean: 8-14 years |
| Jacobs C. *et al.*(54) | 2015 | USA | Retrospective Cohort study | 361 children  237 with functional constipation  48 with chronic abdominal pain  56 with HD  24 post-colon transection | Median: 7.6 years+/-5.1 |
| Queiroz Machado V. *et al.*(80) | 2015 | Brazil | Prospective cohort study | 15 children | Mean: 9.7 years  Range: 7-14 years |
| Quitadamo P. *et al.*(81) | 2015 | UK  Italy | Prospective cohort study | 24 children with chronic constipation refractory to standard treatment | Mean: 9.8 +/- 3.1 years  Range: 4-16 years |
| Ray Parashette KW. *et al.*(82) | 2015 | USA | Prospective cohort study | 13 children | Mean: 9.3 +/- 3.3 years  Range: 4.6-16.2 years |
| Ridha Z. *et al.*(83) | 2015 | Australia | Retrospective case series | 86 children who had undergone nuclear transit studies | Slow Transit Constipation - Median: 71 months; Range: 2-154 months;  Functional Faecal Retention – Median: 60 months; Range: 8-154 months)  Normal Transit: Median: 68.5 months; Range: 14-144 months; |
| Akotia DH. *et al.*(12) | 2016 | USA | Prospective cohort study | 19 neonates | Neonates with no/low peristalsis: 1.6+/-0.3 days old  Neonates with normal/hyperactive peristalsis: 1.9+/-0.6 days old |
| Borrelli O. *et al.*(18) | 2016 | UK | Prospective cohort study | 103 children with slow transit constipation | Median: 8.8 years  Range: 3.2-15.7 years |
| Gomez-Suarez RA. *et al.*(48) | 2016 | USA | Retrospective cohort study | 40 paediatric patients | Mean: 9.5+/-4.4 years |
| Koppen IJN. *et al.*(62) | 2016 | USA, The Netherlands | Cross-sectional survey study | 55 paediatric surgeons and 19 paediatric gastroenterologists assessed via survey | - |
| Stathopoulos L. *et al.*(99) | 2016 | Australia | Retrospective case series | 10 children with persistent faecal incontinence | Mean: 9.6 years  Range: 4.25-15.5 years |
| Wessel S. *et al.*(127) | 2016 | The Netherlands, Australia | Prospective cohort study | 18 children with chronic intractable functional constipation | Median: 15 years  Range: 4–18 years |
| Arbizu RA *et al.*(13) | 2017 | UK | Prospective cohort study | 60 children | Mean age: 9.5 years  Range: 1.5-18-8 years |
| Koppen IJN. *et al.*(60) | 2017 | USA, The Netherlands | Retrospective cohort study | 30 children with functional constipation | Median: 10 years  Range: 8-13 years |
| Daeze C. *et al.*(122) | 2018 | Belgium | Prospective cohort study | 22 children with spina bifida | Median: 7.4 years  Range: 3.2-9.8 years |
| Koppen IJN. *et al.*(61) | 2018 | USA, The Netherlands, Australia | Retrospective cohort study | Intervention: 19 children in the study undergoing solid-state colonic manometry  Control: 18 children from a previous studied cohort who underwent water-perfused colonic manometry | Intervention:  Median: 11.1  Range: 3.9-19.5  Control:  Median: 15 years |
| Sanghavi RM. *et al.*(90) | 2018 | USA | Retrospective case series | 36 children | Range: 3-16 years |
| Shava U. *et al.*(126) | 2018 | India | Prospective case-control study | 100 children:  39 healthy controls (Group A)  61 children with functional constipation (Group B, diagnosed using Rome III criteria). | Healthy controls: median 9 years (range 2.5–17 years)  Functional constipation: median 4.5 years (range 2–14 years) |
| Sintusek P. *et al.*(93) | 2019 | UK, Thailand | Retrospective case-control study | Intervention: 12 children  Control: 8 children | Mean: 10.9 years  Range: 6.5-15 years |
| Dranove J. *et al.*(33) | 2020 | USA | Retrospective cohort study | 34 children investigated with both Oral-anal Transit Test and Colonic Manometry | Mean age: 11.5 years  Range: 4–18 years |
| Heitmann PT. *et al.*(52) | 2020 | Australia, UK, The Netherlands, USA | Retrospective cohort study | 165 children | Median: 10 years  Range: 1-17 years |
| Rodriguez L. *et al.*(87) | 2020 | USA | Retrospective cohort study | 448 children having undertaken 555 studies | Median age: 8.9 years (0.8-24 years) |
| Brinck CE. *et al.*(19) | 2021 | Denmark | Prospective cohort study | 21 children without gastrointestinal symptoms | Mean: 10.8 years  Range: 7-15 years |
| de Sillos MD. *et al.*(31) | 2021 | Brazil | Prospective case series | 22 children with cystic fibrosis | Mean: 8.2 years  Range: 5.7-13 years |
| Rodriguez L. *et al.*(86) | 2021 | USA | Prospective cohort study | 57 children | Median: 16.45 years  Range: 8.78-17.8 years |
| Vriesman MH. *et al.*(112) | 2021 | USA, The Netherlands | Prospective cohort study | 6 children/adolescents with intractable functional constipation | Median: 14 years  Range: 12-17 years |
| Fritz T. *et al.*(41) | 2022 | Germany | Retrospective case series | 9 adolescents with functional gastrointestinal disorders | Median: 15 years  Range: 12-17 years |
| Chen JJ. *et al.*(21) | 2022 | USA | Prospective cohort study | 150 children | Median: 9.9 years  Range: 2.1-18.8 years |
| Infante JR. *et al.*(53) | 2022 | Spain | Retrospective cohort study | 13 children | Range 5-17 years |
| Osatakul S. *et al.*(75) | 2022 | Amsterdam | Prospective cross-sectional survey study | 274 pediatric gastroenterologists | n/a |
| Rodriguez L. *et al.*(85) | 2022 | USA | Retrospective cohort study | 60 children with medically refractory functional constipation | Median: 7.1 years  Range: 0.15-23-6 years |
| Tsai A. *et al.*(103) | 2022 | USA | Retrospective case series | 436 abdominal radiographs from paediatric patients | Mean: 12.1 +/- 4.3 years  Range: 3-21 years |
| Coe A. *et al.*(27) | 2023 | USA | Retrospective case-control study | Cases: 56 children with constipation and ASD  Controls: 123 children with constipation but not ASD | Cases:  Mean: 10.7+/-4.07  Range: 2-18.3  Controls:  Mean: 10+/-5.27  Range: 1.8-23.6 years |
| Colliard K. *et al.*(28) | 2023 | UK | Retrospective case series | 445 children | Median: 9 years  Range: 0.8-19 years |
| Dorfman L. *et al.*(38) | 2023 | USA | Retrospective cohort study | 89 children with idiopathic constipation and reported normal colonic manometry | Median: 9 years (5-12 years) |
| Jacobs SE. *et al.*(55) | 2023 | USA | Retrospective case series | 22 children with colonic motility disorder refractory to medical management | Median: 10 years  Range 6-21 years |
| Mansi S. *et al.*(69) | 2023 | USA | Prospective cohort study | 45 children:  22 received bisacodyl  23 received glycerin | Median: 10 years  Range: 2-18 years |
| Mehta V. *et al.*(72) | 2023 | USA | Prospective cohort study | 38 Children  16 only included in final analysis | Mean: 11+/-3 years  Range: 4-18 years |
| Yuan Y. *et al.*(115) | 2023 | China | Prospective cohort study | 30 children with refractory constipation | Range: 2-18 years |
| Dorfman L. *et al.*(35) | 2024 | USA | Retrospective cohort study | 68 colonic manometry studies in children | Median: 10.3 years (5.1-14.5 years) |
| Dorfman L. *et al.*(34) | 2024 | USA | Retrospective cohort study | 20 children with medically refractory idiopathic constipation | Median: 12 years (8-15 years) |
| Fu Y. *et al.*(42) | 2024 | USA | Retrospective cohort study | 30 children post-pull through for Hirschsprung Disease | Median: 7 years  Range: 17 months – 21 years |
| Rolleman N. *et al.*(89) | 2024 | The Netherlands | Prospective cohort study | 20 participants  Paediatrics: 9 intervention, 7 control  Adults: 2 intervention, 2 control | Paediatric cohort:  Intervention: 1.5 years (0.5-5 years)  Control: 0.3 years (0.3-0.4 years) |
| van der Zande JS. *et al.*(105) | 2024 | Netherlands, USA | Retrospective case-control study | Tethered cord syndrome group: 24 children  Control group: 24 age-matched children with faecal incontinence | Median: 7.5 years (5-10.3 years) |
| Chanpong A. *et al.*(5) | 2025 | UK | Prospective cohort study | 22 children with constipation referred to Great Ormond Street Hospital and Queensland Children’s Hospital for further investigation | Median age: 10.94 years |
| Dorfman L. *et al.*(37) | 2025 | USA | Retrospective cohort study | 443 colonic manometry studies in children | Median: 9 years (5.4-13.6 years) |
| Dorfman L. *et al.*(36) | 2025 | USA | Cross-sectional survey study | 37 paediatric motility centres | - |
| Shan AYC. *et al.*(91) | 2025 | USA | Retrospective cohort study | 13 children with myelomeningocele | Median: 7 years (5-11 years) |

## **Appendix 2 - Table 2:** Summary table of papers comparing colonic motility investigations.

| **Author** | **Colonic motility investigation modalities in children** | **Motility Investigation Advantages** | **Motility Investigation Disadvantages** |
| --- | --- | --- | --- |
| 1996 – Staiano A. *et al.*(97) | 1) Radiopaque Markers; | 1) Objective measurement of transit; Non-invasive; | 1) Radiation exposure; |
| 1997 – Pigeon N. *et al.*(79) | 1) Radiopaque Markers;  2) Anorectal manometry; | 1) Assessment of total and segmental transit time; Identification of different patterns of constipation with segmental localisation;  2) Objective assessment of anorectal function; | 1) Variability in results according to methodology and reference values; Radiation exposure; Requires patient compliance;  2) Needs to be used as a test in adjunction to others for clinical use; |
| 1997 – Rintala RJ. *et al.*(84) | 1) Radiopaque Markers;  2) Contrast enema; | 1) Simple; Cheap; Assessment of both total and segmental transit; Validated;  2) Anatomical assessment; Useful for longitudinal follow-up; | 1) Compliance required; Indirect assessment of motility;  2) Structural information only, no motility information; |
| 1998 – Hamid SA. *et al.*(124) | Colonic manometry | Differentiation between functional faecal retention and chronic intestinal pseudo-obstruction; High-diagnostic accuracy; | Expensive; Invasive; Need for further investigation and use in children to draw clinical conclusions; |
| 1998 – Shankar KR. *et al.*(106) | 1) Radiopaque Markers;  2) Anorectal manometry; | 1) Simple; Assessment of the whole colon;  2) Direct assessment of sphincter function; | 1) Radiation exposure;  2) Invasive; |
| 1999 – Baillie CT. *et al.*(15) | Radiopaque Markers; | Simple protocol; | Ingestion of 10 radiopaque markers for 6 consecutive days – difficulty swallowing in children, risk of forgetting; Dietary restriction for days of study; Approximation of motility counting markers on AXR; Radiation exposure; |
| 1999 – Chung KS. *et al.*(23) | Radiopaque Markers; | Available healthy children reference data; | Inability to swallow capsule; Radiation exposure; |
| 1999 – Halabi IM. *et al.*(123) | Carmine marker; | Objective measure of gastrointestinal7colonic transit; Simple; Inexpensive; | No segmental colonic transit information; Repeated tool collection required; Patient compliance; |
| 1999 – Heikenen JB. *et al.*(51) | 1) Colonic manometry (water perfused catheters);  2) Anorectal manometry; | 1) Helpful in guiding clinical management;  2) Complements colonic manometry providing information about sphincter function; | 1) Invasive; General anaesthetic required for colonoscopy; Radiation exposure; Need for specialised equipment and expertise;  2) Requires patient cooperation for full assessment; Invasive; |
| 1999 – Ure BM. *et al.*(104) | Radiopaque Markers; | Objective assessment of bowel transit; | Variable protocols; Challenges in swallowing markers in paediatrics; Limited paediatric normative data; Radiation exposure; |
| 1999 – Wheatly JM. *et al.*(128) | Scintigraphy; | Objective assessment of whole-gut and colonic transit; | No direct assessment of colonic motor physiology; |
| 2000 – Di Lorenzo C. *et al.*(32) | 1) Colonic Manometry;  2) Anorectal Manometry;  3) Barium enema; | 1) Colonic motility assessment during fasting, post-prandial and post-chemical stimulation phase; Classification of four colonic motility subgroups;  2) Internal anal sphincter assessment;  3) Assessment for anatomical strictures; | 1) General anaesthetic for catheter placement via colonoscopy; Radiation exposure; Hospital admission; |
| 2000 – Lucanto C. *et al.*(68) | 1) Colonic manometry;  2) Anorectal manometry; | 1) Differentiation between colonic neuropathy and colonic myopathy;  2) Identification of anorectal causes for constipation; | 1) Invasive; Limited recording duration; Required specialised expertise and equipment; |
| 2000 – Miele E. *et al.*(73) | 1) Radiopaque Markers;  2) Anorectal manometry; | 1) Localisation of transit delay; Results able to be compared with controls data;  2) Objective assessment of anorectal function; | 1) Potential difficulty in interpretation of results if anatomical variations; Limited precise pathophysiologic interpretation;  2) Normal anorectal results may coexist with colonic dysmotility; |
| 2001 – Guimaraes EV. *et al.*(49) | 1) Radiopaque markers;  2) Anorectal manometry; | 1) Able to establish transit time; Validated methodology;  2) Helpful in diagnosis of Hirschsprung Disease; | 1) Radiation exposure; Patient compliance and follow-up required; No assessment of colonic contractility; Protocol variability in different centres;  2) Invasive; Requires patient collaboration and in paediatrics it might be challenging requiring the patient to be asleep for the investigation; |
| 2001 – Treepongkaruna S. *et al.*(102) | 1) Scintigraphy;  2) Anorectal manometry; | 1) Whole gut and segmental transit information; Non-invasive physiological assessment; | 1) Lack of paediatric control data; |
| 2001 – Villarreal J. et al.(111) | Colonic manometry; | Identification of segmental disease; | Data interpretation challenges in severely dilated colon; Invasive; |
| 2002 – Gutierrez C. *at al.*(46) | 1) Radiopaque Markers;  2) Radioactive Techniques;  3) Breath tests;  4) Anorectal Manometry; | 1) Simple; Non-invasive;  2)   3) Non-invasive; | 1) Need to be able to swallow capsules; Risk of reduced compliance; Differences in protocols; Radiation exposure;  2) Difficult to perform and require selective intubation of the digestive tract;  3) Orocaecal transit time, which is of little importance in the physiopathology of constipation; |
| 2002 – Shin YM. *et al.*(92) | Scintigraphy; | Objective assessment of whole gut transit and colonic transit; | Exposure to radioisotopes; Specialised and resource-intense; Results interpretation variability according to institution; |
| 2003 – Pensabene L. *et al.*(78) | Colonic manometry; | Distinguish between functional constipation and colonic neuromuscular disorders; Perceived benefit of investigation from families; | Invasive; Requires sedation/anaesthesia; Specialised staff and equipment; Difficult results interpretation in dilated colon; Risk for artifacts; Resource-intensive; |
| 2004 – Benninga MA. *et al.*(16) | 1) Radiopaque Markers;  2) Anorectal manometry; | 1) Simple protocol; | 1) Radiation exposure; Availability of comparable healthy control values; Inability to swallow capsules; Mathematically-surrogate estimation of segmental colonic transit/motility;  2) Enema disimpaction required days priors; Patient awake and following commands to have full exam results; |
| 2004 – Chitkara DK. et al.(22) | 1) Anorectal manometry;  2) Scintigraphy; | 1)   2) Non-invasive; | 1) Insertion of catheter per rectum;  2) Overnight fast; Mathematical calculations for transit, specialised centres expertise; |
| 2004 – De Lorijn F. *et al.*(29) | Radiopaque Markers; | Differentiation between retentive and non-retentive defaecation disorders; | Inability to swallow capsules; Radiation exposure; |
| 2004 – Langer J. *et al.*(63) | 1) Radiopaque Markers:  2) Colonic Manometry;  3) Anorectal Manometry; | 1) Useful screening investigation;  2) Description of aetiologies for postoperative obstruction;  3) Objective sphincter function assessment; | 1) Cannot define type and extent of dysmotility; Results may be affected by faecal impaction;  2) Limited availability; Lack of standardisation; Specialised expertise required;  3) Limited value in identifying the cause of obstruction; |
| 2004 – Martin MJ. *et al.*(70) | 1) Colonic manometry; | 1) Measurement of colonic intralaminar pressure/contractions; Identification of dysmotile segments; Safe test with no complication in this paper; | 1) Specialised and invasive investigation; Limited availability; Assessment of the colon only; Lack of control reference values; |
| 2004 – Park ES. *et al.*(76) | Radiopaque Markers; | Objective measurement of constipation severity; Segmental transit assessment; Simple technique; | Lack of exact identification of pathophysiology for constipation; Radiation exposure |
| 2004 – Wagener S. *et al.*(113) | Radiopaque Markers; | Measurement of both total and segmental transit; Well-tolerated; Simple protocol; | Variation in normal reference values according to different protocols; Radiation exposure; |
| 2004 – Zaslavsky C. *et al.*(117) | Radiopaque Markers; | Objective measure of colonic function; Assessment of total and segmental transit; Simple and validated method; | Radiation exposure; Requires patient compliance; Transit information but no direct motility information; |
| 2005 – Gertken JT. *et al.*(43) | Colonic manometry; | Differentiation between functional faecal retention and colonic neuromuscular disease; Tool that can graphically support patients/families’ education; | Invasive; Psychological distress; Resource-intense; |
| 2005 – Soares ACF. *et al.*(94) | 1) Radiopaque Markers;  2) Breath methane test; | 1) Simple; Low radiation protocol;  2) Non-invasive; Simple and well tolerated; | 1) Lack of description of underlying mechanism for dysmotility; Compliance; Radiation exposure;  2) Indirect measure of colonic function; Influenced by intestinal microbiology; |
| 2005 – Stanton MP. *et al.*(98) | Colonic manometry (24-hour); | Assessment of the entire colon for direct motor function; | Invasive and specialised; Limited availability; Technical complexity; |
| 2005 – Watanabe Y. *et al.*(114) | Water-Selective Extraction MR - Cine Imaging; | Assessment of the whole gastrointestinal tract simultaneously; No ionising radiation; Non-invasive; No breath-holding required; assessment of both anatomy and function; Pre- and post-prandial motility assessment; | Qualitative; Clinical diagnostic value not established yet; Limited in very young children; MRI availability and expertise required; |
| 2005 – Zhang SC. *et al.*(118) | 1) Radiopaque Markers;  2) Defaecography;  3) Contrast enema; | 1) Objective assessment of colonic transit; Simple standardised method;  2) Dynamic anorectal function result;  3) Anatomical detail; | 1) Radiation exposure; Indirect measurement of motility;  2) Radiation exposure; Invasive and uncomfortable;  3) Radiation exposure; |
| 2006 – Zuccarello B. *et al*.(121) | Radioisotope Scintigraphy; | Identification of the 2 subtypes of colonic dysmotility (slow-transit constipation and functional rectosigmoid obstruction); Provision of more details and greater sensitivity than solid makers studies; |  |
| 2007 – Bijos A. *et al.*(17) | 1) Ultrasonography;  2) Radiopaque Markers; | 1) Simple methods; Measurement of transverse diameter of rectal ampulla and assessment of stool retention; If megarectum identified, referral for colonic transit;  2) Simple protocol; | 1) Need for several reattempts at measurement to ensure accuracy;  2) Radiation exposure; Mathematically-surrogate estimation of segmental colonic transit/motility; |
| 2007 – Kohno M. *et al.*(60) | Colonic manometry (lower colon only) | Colonic neuromuscular assessment; | Limited distal colon contractility information if lower colonic manometry only; Technical difficulties of recording during defaecation; |
| 2007 – Zhang SC. *et al.*(119) | 1) Radiopaque Marker;  2) Defaecography;  3) Contrast enema; | 1) Objective assessment of transit;  2) Information about anorectal mechanics continuing to soiling and incontinence;  3) Anatomical information; | 1) Radiation exposure; No pathophysiology information;  2) Radiation exposure; Invasive and uncomfortable;  3) Radiation exposure; |
| 2008 – Venkatasubramani N. *et al.*(110) | Colonic manometry; | Direct assessment of colonic motor activity; | Invasive procedure; Requirement for specialised equipment and general anaesthesia; |
| 2008 – Demirogullari BO. *et al.*(30) | 1) Radiopaque Markers;  2) Barium enema; | 1) Total and segmental colonic transit assessment;  2) Colonic anatomy; Establish presence of megarectum; | 1) Inability to swallow markers; Radiation exposure;  2) Radiation exposure; Anatomical rather than functional information: Not able to assess severity of constipation; |
| 2008 – King SK. *et al.*(58) | Colonic manometry (24hr ambulatory manometry with water-perfused catheter) | Evaluation of colon during different phases; | No healthy paediatric control group; Bowel preparation may affect results; Measurement of pressure activity rather than actual transit/flow; Unsure if reflect actual daily colon physiology as tested during an inpatient controlled environment; |
| 2008 – Yuan Z. *et al.*(116) | 1) Anorectal manometry;  2) Radiopaque Markers; | 1) Objective assessment of anorectal dysfunction; Measures treatment response;  2) Objective assessment of colonic transit; | 1) Invasive specialised investigation;  2) Requirement for repeated marker ingestion and radiography; Transit information only; |
| 2009 - Aspirot A *et al.*(14) | Colonic manometry; | Colonic motility information in fasted state, post-prandial state and post-chemical stimulation; Guidance with patient selection for successful anterograde enema management; | Bowel washout; Colonic catheter placement; radiation exposure for catheter position confirmation; |
| 2009 – Clarke MC. *et al.*(26) | Scintigraphy; | Total colonic transit information and segmental colonic transit information to differentiate between slow transit and chronic intestinal outlet obstruction; | Radiation exposure; Mathematical extrapolation of transit; |
| 2009 – Clarke MMC. *et al.*(25) | Scintigraphy; | Gastric and colonic emptying assessment; Differentiation between slow transit, anal retention and normal transit; Accurate assessment of regional colonic transit with multiple images of the colon being easily obtained with a low radiation dose; No concerns for overlapping regions of the gastrointestinal tract; More physiological transit results than with radiopaque markers as these are indigestible; | Radiation exposure; No healthy reference ranges in paediatrics; |
| 2009 – Soares ACF. *et al.*(95) | Radiopaque Markers; | Objective assessment of colonic transit; | Radiation exposure; Multiple days of testing; |
| 2009 – Sutcliffe JR. *et al.*(100) | Scintigraphy; | Identification of site of hold-up; Assessment of whole-gut transit; Reproducible; | Radiation exposure; Specialised expertise required; |
| 2010 – Dranove R. *et al.*(4) | Colonic manometry; | Provision of objective evidence of colonic motility; Assessment for underlying neuropathy or myopathy; Guidance for further therapeutic intervention; Qualitative and quantitative motility measurement; | Invasive; Expensive; |
| 2010 – Gomez R. *et al.*(45) | Colonic manometry; | Objective data about colonic motor activity; Able to compare colonic response to different stimuli; | Invasive; Bowel preparation required; Specialised equipment and expertise; |
| 2010 – Koh HL. *et al.*(59) | Radiopaque Markers; | Assessment of colonic transit; Distinction between colonic dysfunction and distal obstruction/outlet dysfunction; | Time consuming; Radiation exposure with more than one Xray being performed; |
| 2010 – Liem OB. *et al.*(67) | Colonic manometry; | Assessment of colonic neuromuscular response to fasting, meal and chemical challenge with bisacodyl; | Technical challenges; Challenges in standardisation of catheter placement; Invasive; Patient discomfort; No paediatric normative data; |
| 2010 – Märzheuser S. *et al.* (71) | 1) Hydrocolonic sonography; | 1) Usefulness in displaying the width of bowel loops and evaluating colonic peristalsis; Lack of radiation exposure; Limited invasiveness; Reduced costs; | 1) Variable accuracy depending on the indications and study population; |
| 2011 – Grasshoff-Derr S. *et al.*(47) | Hydrocolonic ultrasonography; | Determination of stool texture, activity of the colon, appropriate volume needed to fill up and clean the colon;  Less invasive diagnostic tool as an alternative to barium contrast enemas and colonoscopy for diagnostic purposes of the colon and rectum;  Absence of radiation;  Available in most centres;  Tolerated at all ages;  Takes only 10–30 min of time and is  less invasive and non-painful;  Frequently reported problems are immediately visible;  Valuable tool for a long-term follow-up and can help to  differentiate the underlying problem;  Identification of most optimal adapted volume of the enema, and  content of the enema based on the characteristics of the colon; |  |
| 2011 – Kaul A. *et al.*(56) | 1) Colonic manometry (water perfused catheters);  2) Anorectal manometry; | 1) Able to physiologically identify different colonic motility groups;  2) Objective assessment of anal sphincter function; | 1) Lack of normative paediatric values; Invasive; Need for specialised equipment and staff;  2) Invasive; Usually needs pairing with colonic manometry; |
| 2011 – van der Baan-Slootweg JS. *et al.*(105) | Radiopaque Markers; | Non-invasive; Toal and segmental transit time information; | Radiation exposure; |
| 2012 – Clarke MCC. *et al.*(24) | Colonic manometry (24hr study with water-perfused catheters); | Assessment of colonic contractility | Radiation exposure |
| 2012 – Liem O. *et al.*(66) | Colonic manometry; | Solid-state catheters allow ambulation and might be more sensitive than water-perfused catheters; Solid-state no risk of water overload; | Technical difficulties in particular with water-perfused catheters; Sensitivity of test decreases with colonic distension; High cost of solid-state catheters; Maintenance and fragility of equipment; |
| 2012 – Rodriguez L. *et al.*(108) | 1) Colonic manometry;  2) Anorectal manometry; | 1) Direct colon motor activity assessment;  2)  | 1) Limited assessment with no high-resolution catheters; Potential for movement artifact; Invasive;  2) Sedation may affect results; |
| 2012 – Tipnis NA. *et al.*(101) | 1) Radiopaque Markers;  2) Colonic manometry (water-perfused system); | 1) Simple, office-based investigation;  2) Information about whole colonic and segmental colonic transit assessing for neuropathic or myopathic disease; | 1) Poor information about segmental colonic transit; Protocol variation; Variation in normal values; Need to swallow markers (limitation for young children, children with dysphagia or neurological impairment); Radiation exposure;  2) Bowel preparation; Need for general anaesthesia and hospital admission; |
| 2012 – Yik YI. *et al.*(129) | Scintigraphy; | Objective assessment of colonic transit; | No explanation for underlying pathophysiological mechanism; |
| 2013 – Firestone Baum C. *et al.*(40) | Colonic manometry; | Differentiation between functional constipation and neuromuscular disease; Useful biofeedback tool; Useful to identify behavioural and psychosocial contributors; | Hospital admission as test requires time; Expertise required; Invasive; Stressful for children; |
| 2013 – Giorgio V. *et al.*(44) | Colonic manometry; | High-resolution manometry detects abnormalities missed by conventional manometry; May expedite diagnosis of colonic neuropathy and surgical management; | Invasive; No healthy control data available in paediatrics; |
| 2013 – Hedsund C. *et al.*(50) | 1) Radiopaque markers;  2) Scintigraphy;  3) Motility Tracking System (magnetic tracking studies with high resolution);  4) Wireless Motility Capsule; | 1) Protocols easy to follow and possibility to draw ambulant estimation of gastrointestinal transit and segment colorectal transit times;  2) Precise information about gastric emptying, small intestine transit time and segmental colorectal time;  3) Minimally invasive; Radiation-free method;  4) Ambulatory assessment method; | 1) Exposure to radiation; Lack of information about gastric emptying and small intestinal transit time;  2) Expensive; not readily available; radiation exposure;  3) Stationary recording; Participants need to be able to swallow the pill; Standardized size of the pill to be injected despite children having smaller anatomy than adults and at different ages in amongst themselves;  4) No assessment of segmental colonic transit time nor quantification of colonic mass movements; No data in children; |
| 2013 – Keshtgar AS. *et al.*(57) | 1) Anorectal manometry;  2) Anorectal endosonography;  3) Radiopaque Markers; | 1) Objective assessment; Helpful in diagnosis of HD;  2) Structural assessment of anal sphincter;  3) Objective assessment of colonic transit; Simple and widely available; | 1) Need for sedation, therefore limited assessment;  2) No assessment of sphincter function; Need for sedation in paediatrics; Specialised equipment and expertise;  3) Radiation exposure; Patient compliance over days required; |
| 2013 – Marte A. *et al.*(125) | Radiopaque Markers; | Objective assessment; Simple; | Radiation exposure; No colonic motor pattern assessment; |
| 2013 – Mugie SM. *et al.*(74) | 1) Colonic manometry;  2) Scintigraphy; | 1) Characterisation of colonic motor function; Total colonic and segmental information; Able to test outcomes of chemical stimulation; Evaluation of behaviour around defaecation;  2) Non-invasive and well tolerated; Objective assessment of colonic transit; Useful screening tool to them proceed to further colonic motility testing if needed; Low radiation exposure; | 1) Invasive; Requires anaesthesia/sedation; Technically challenging and limited availability, requiring specialised staff and hospitalisation; Time-consuming test; Poor patient preference outcome for the test;  2) Expensive; Potential capsule malfunction; Difficulty swallowing capsule; Potential for false abnormal result; |
| 2013 – Pedersen CE. *et al.*(77) | Radiopaque Markers; | Segmental transit analysis; Established and validated technique; | Radiation exposure; Difficulties with compliance; Variation in protocols and data interpretation; Transit information only; |
| 2013 – Rodriguez L. et al.(88) | Colonic manometry; | Assessment of physiological motor patterns; | Invasive; |
| 2013 – Velde SV. *et al.*(107) | 1) Radiopaque Markers;  2) Anorectal manometry; | 1) Assessment of both total and segmental colonic transit; Availability of control comparative values; | 1) Markers may not behave like stool; No assessment of slow intestine; Analysis may be affected by anatomy;  2) Invasive; |
| 2013 – Velde SV. *et al.*(109) | Radiopaque Markers; | Non-invasive and simple; Widely available; | Markers may not behave like stool; Radiation exposure; |
| 2014 – El-Chammas KI *et al.*(39) | Colonic manometry (solid-state catheters); | Distinguish functional constipation from colonic neuromuscular disorders; Assistance in prediction of successful surgical management for constipation; No risk of fluid overload with solid-state catheters; | Variability in data analysis and reporting; Movement may shift sensors location; Bowel preparation; General anaesthetic; Colonoscopy/fluoroscopy required; |
| 2014 – Kerur B. *et al.*(64) | 1) Colonic Manometry;  2) Defecography; | 1) Preferred test for differentiating children with anorectal retention from those with slow colonic transit;  2) Evaluation of both morphological and dynamic factors of defecation; More physiological positioning of the patient while imaging is taken, impacting less on pelvic floor physiology and dynamics of defaecation; | 1)   2) Clinical significance is debateable; |
| 2014 – Liem O. *et al.*(65) | Colonic manometry (solid-state catheters); | Solid state catheters allow ambulatory movement during testing; | Results can vary depending on study length; Risk of catheter dislodgement and technical challenges; Lack of paediatric normative data; |
| 2014 – Zivkovic VD. *et al.*(120) | Scintigraphy; | Information about different constipation subtypes; Low radiation dose; Information about whole and segmental gastrointestinal motility; | Specialised equipment and expertise required; Radiation exposure; Requirement for multiple visits/images; |
| 2015 – Carmo RLML. *et al.*(20) | 1) Anorectal manometry;  2) Barium enema;  3) Nuclear transit study; | 1)   2)   3) Non-invasive; Whole gastrointestinal assessment as well as segmental assessment; | 1)  Rectal disimpaction prior;  2)   3) Hospital admission; Rectal disimpaction prior; |
| 2015 – Jacobs C. *et al.*(54) | Colonic manometry; | Objective assessment of colonic neuromuscular function; | Invasive; Resource-intensive; Lack of normative paediatric controls; Results may be affected by general anaesthetic and bowel preparation; |
| 2015 – Queiroz Machado VM. *et al.*(80) | Radiopaque Markers; | Quantitative measurement of total and segmental colonic transit; Identification of segmental localisation of transit delay; Possibility to use radiation minimisation measures; | Radiation exposure; Multi-day testing required; No identification of the cause for dysmotility; |
| 2015 – Quitadamo P. *et al.*(81) | Radiopaque Markers; | Cheap; Readily available; Easy to interpret; Segmental and total transit assessment; | Influenced by faecal loading; Radiation exposure; |
| 2015 – Ray Parashette KW. *et al.*(82) | Colonic manometry; | Quantitative assessment of colonic motor activity; Evaluation of colonic response to chemical stimulation; | Invasive; Requires anaesthesia/sedation; Lack of normative paediatric data; |
| 2015 – Ridha Z. *et al.*(83) | Scintigraphy; | Information about transit time and tracer distribution; | Expensive; Time-consuming; Need for patient compliance; |
| 2016 – Akotia DH. *et al.*(12) | 1) Ultrasonography; | 1) Non-invasive | 1) Lack of normal motility reference values |
| 2016 – Borrelli O. *et al.*(18) | 1) Radiopaque markers;  2) Colonic manometry; | 1) Simple protocol;  2)  | 1) Radiation exposure; Mathematically-surrogate estimation of segmental colonic transit/motility;  2) Semiliquid diet prior to the investigation and then fasting ahead of general anaesthetic; Bowel washout; Radiation exposure (one AXR); Long time for investigation assessment; Absence of reference values; |
| 2016 – Gomez-Suarez RA. *et al.*(48) | 1) Colonic manometry;  2) Anorectal manometry; | 1) Objective assessment of colonic contractility; Assessment of colonic response to meal and bisacodyl;  2) Objective assessment of anorectal function; Complementary information to colonic manometry; | 1) Invasive; requires general anaesthetic and colonoscopy for catheter placement; Specialised equipment and expertise needed;  2) Limited predictive value for appendicostomy success; Patient cooperation required for full assessment; Invasive and uncomfortable; |
| 2016 – Koppen IJN. *et al.*(62) | 1) Colonic manometry;  2) Anorectal manometry; | 1) Guidance in surgical management of children with constipation; Distinguish between organic and functional disease; Identification of dysmotile segments;  2) Guidance in diagnosis of HD; Objective assessment of anal sphincter function and integrity; | 1) No clear evidence-based guidelines; Variable results interpretation;  2) Variable consensus in amongst specialist regarding clinical interpretation of results; |
| 2016 – Stathopoulos L. *et al.*(99) | 1) Scintigraphy;  2) Breath test; | 1) Assessment of whole-gut and colonic transit; Non-invasive;  2) Non-invasive; Able to detect sugar malabsorption; | 1) Radiation exposure; Assessment of transit only; Specialised centres;  2) Positive results do not prove causation; Debated clinical relevance of a positive breath test; |
| 2016 – Wessel A. *et al.*(127) | Colonic manometry (high resolution); | Detailed characterization of multiple colonic motor patterns; Can help identify children with suspected colonic neuropathy; | Invasive; Technically complex and time-consuming; Specialised equipment and staff; |
| 2017 – Arbizu RA. *et al.*(13) | Colonic manometry; | Motility assessment during fasting state, post-prandial state and post-chemical stimulation state; | Hospital admission; Bowel preparation; General anaesthetic for colonoscopy to insert catheter; Altered interpretation of results due to general anaesthetic effect; |
| 2017 – Koppen IJN. *et al.*(60) | Colonic manometry (solid state catheters); | Solid-state catheters comparable to water-perfused catheters in terms of findings; | Lack of standardisation of protocols, equipment, analysis techniques; Risk to miss propagating cyclic motor patters and short-single motor patters with sensors spaced at 3cm; Limited normative comparative data; Invasive; Bowel preparation; |
| 2018 – Daeze C. *et al.*(122) | 1) Radiopaque Markers;  2) Anorectal manometry; | 1) Assistance with surgical planning; Non-invasive;  2) Useful to assess sphincter function in patients with symptoms but normal colonic transit time; | 1) Markers ingestion; Radiation exposure;  2) Invasive; Time-consuming; Stressful for children; |
| 2018 – Koppen IJN. *et al.*(61) | Colonic manometry (solid state catheters); | Solid-state catheters comparable to water-perfused catheters in terms of findings; | Lack of standardisation across centres; Invasive; Catheter-type dependent detection of motor patterns; No paediatric control data; Bowel preparation; |
| 2018 – Sanghavi RM. *et al.*(90) | Colonic manometry; | Objective assessment of colonic function; | Technically challenging; Time-consuming; Requirement for colonoscopy and fluoroscopy; Need for anaesthesia; |
| 2018 – Shava U. *et al.*(126) | Radiopaque Markers; | Objective assessment of total and segmental colonic transit time; | Radiation exposure; |
| 2019 – Sintusek RM. *et al.*(93) | 1) Colonic manometry;  2) Radiopaque Markers;  3) Anorectal manometry; | 1) Direct assessment of colonic motor function;  2) Useful screening tool;  3) Objective anal sphincter assessment; | 1) Invasive; Data could be influenced by anaesthesia and timing of testing; Technically challenging; Resources-intensive;  2) Assessment of transit only; Radiation exposure;  3) Invasive; |
| 2020 – Dranove J. *et al.*(33) | 1) Radio-opaque Markers;  2) Colonic Manometry; | 1) Simple;  2) Well-tolerated;  3) Reduced amount of radiation if only one Xray; | 1) Variation in protocols; Radiation exposure;  2) Bowel preparation required; Need for general anaesthesia; Limited availability; Expensive; Invasive; |
| 2020 – Heitmann PT. *et al.*(52) | Colonic manometry; | Assessment of colonic neuromuscular function; Guidance in surgical management decision-making for constipation; Findings reproducible across different centres; | Diagnostic value still undetermined; Limited availability of test; Invasive; lack of normative paediatric values; |
| 2020 – Rodriguez L. *et al.*(87) | 1) Colonic manometry; | 1) Useful investigation in predicting response to surgical interventions and the eventual need for surgery; | 1) Challenges with completion of meal stimulus part of the investigation for patients with severe feeding intolerance; Colonic manometry not predictive of medical changes in management; |
| 2021 – Brinck CE. *et al.*(19) | 3D-Transit system (small electromagnetic capsule); | Ambulatory assessment in own environment; No dietary restrictions during study; Non-invasive; Whole gut and segmental motility information; | Challenges in swallowing capsule in paediatrics; Fasting required; three hospital visits required; Manual analysis; Non-direct measurement of gastrointestinal contractions; Minor restrictions in daily activities (ie. sports); No FDA approval; |
| 2021 – de Sillos MD. *et al.*(31) | Radiopaque Markers; | Measurement of total and segmental colonic transit; Assessment for faecal impaction; | Inability to swallow capsules; Administration of enema if faeculoma present; Radiation exposure; Risk of inadequate technical quality of abdominal XRays; |
| 2021 – Rodriguez L. *et al.*(86) | 1) Wireless Motility Capsule;  2) Radiopaque Markers; | 1) Assessment of whole gut transit time; Minimally invasive; Provision of transit and motility information; Well tolerated by patients;  2) Established reference standard, validated in the paediatric population; | 1) Difficulty swallowing capsule; Risk of capsule retention; No paediatric normative values;  2) Transit assessment only; Radiation exposure; |
| 2021 – Vriesman MH. *et al.*(112) | 1) Cine-MRI;  2) Colonic manometry; | 1) Non-invasive; No bowel preparation nor anaesthesia required; Available at different centres; Direct visualisation of colonic wall motion; Shorter investigation than colonic manometry;  2) Differentiation of normal motility from neuromuscular disorders; Objective assessment of intraluminal pressure; | 1) Claustrophobia; Underestimation of motility; Lack of normative paediatric data; Still an experimental research tool;  2) Invasive; Requirement for bowel preparation; Requirement for sedation/general anaesthesia; Expensive and resource-intensive; Limited availability; Recording during non-physiological testing conditions; |
| 2022 – Fritz TH. *et al.*(41) | Wireless Motility Capsule; | Non-invasive; whole gastrointestinal tract assessment; No radiation exposure; Transit and contractility information; No capsule retention in adolescents; | Single pressure sensor; Lack of paediatric reference values; No FDA approval; Inability to swallow capsules; |
| 2022 – Infante JR. *et al.*(53) | Scintigraphy; | Evaluation of the whole gastrointestinal tract with total and segmental transit information; Non-invasive; Helps guide potential benefit of surgical management for constipation; | Prolonged imaging over multiple days; Radiation exposure; Available only at specialised centres; |
| 2022 – Chen JJ. *et al.*(21) | 1) Colonic manometry  (water-perfused catheters or solid-state catheters); | 1) Motility assessment during fasted state, post-prandial state, post-chemical stimulation state; | 1) Inpatient bowel washout; general anaesthesia for catheter placement via colonoscopy; |
| 2022 – Osatakul S. *et al.*(75) | 1) Radio-opaque Markers;  2) Colonic Scintigraphy;  3) Colonic Manometry; | 1) Simple, cheap, readily available; | 1) No provision of specific transit time of each colonic region; |
| 2022 – Rodriguez L. *et al.*(85) | 1) Colonic manometry;  2) Anorectal manometry;  3) Contrast enema; | 1) Guidance for surgical decision-making;  2) Assessment of anal sphincter function;  3) Assessment of colonic anatomy; | 1) Invasive; Specialised investigation;  2) No functional assessment; |
| 2022 – Tsai A. *et al.*(103) | Radiopaque Markers; | Readily available and validated investigation; Simple; | “Tedious” and time-consuming analysis; Radiation exposure; |
| 2023 – Coe A. *et al.*(27) | Colonic manometry (water perfused and solid-state catheters); | Colonic motility recording in fasted, post-prandial and post-chemical stimulation phases; | General anaesthetic catheter placement via colonoscopy or interventional radiology; Radiation exposure; Hospital admission for 2-day study at times; |
| 2023 – Colliard K. *et al.*(28) | Colonic manometry (water perfused catheters); | Colonic motility recording in fasted, post-prandial and post-chemical stimulation phases; | No healthy controls data available; General anaesthetic placement of catheters via colonoscopy; Potential confounders: colonic distension, type and duration of general anaesthetic, efficiency of bowel preparation; |
| 2023 – Dorfman L. *et al.*(38) | Colonic manometry; | Ability to identify gastrocolonic response to visual/olfactory stimuli via visual interpretation of data and motility index; | Visual analysis-only of data can be inaccurate; General anaesthetic; Colonoscopic insertion of catheter; Lack of control paediatric values; |
| 2023 – Jacobs SE. *et al.*(55) | 1) Colonic manometry;  2) Anorectal manometry; | 1) Guidance regarding surgical management for children with colonic dysmotility;  2) Helpful in diagnosis of HD; Objective sphincter assessment; | 1) Limited availability of test at specific centres requiring specialised staff and equipment; Invasive;  2) Specialised expertise required; Invasive; |
| 2023 – Mansi S. *et al.*(69) | 1) Colonic manometry;  2) Anorectal manometry; | 1) Assessment of segmental and whole colonic motility; Evaluation of colonic response to pharmacological stimulation;  2) Objective assessment of anorectal function; Assessment for distal outlet dysfunction; | 1) Invasive; Risk of catheter displacement compromising studies; Need for specialised equipment and expertise; Time consuming protocol; |
| 2023 – Mehta V. *et al.*(72) | Colonic manometry; | Direct assessment of colonic contractility; Quantitative analysis of motility; | Invasive; Technically demanding; Potential confounding factors from general anaesthetic; Lack of healthy control data; |
| 2023 – Yuan T. *et al.*(115) | Colonic manometry; | Information about pathophysiology; Evaluation of meal-induced gastrocolic response; | Invasive; Long-study duration; Specialised equipment and expertise; Requirement for anaesthesia; Lack of healthy paediatric controls; Complex interpretation; |
| 2024 – Dorfman L. *et al.*(35) | Colonic manometry (water perfused and solid-state catheters); | Total colonic and segmental colonic motility assessment; Help guidance if success of surgical management for constipation; Clinically important information even if catheter just in/dislodges to left colon; | Invasive; General anaesthesia; Hospital admission; Dilated colon with redundant colon making catheter placement challenging; Expensive; |
| 2024 – Dorfman L. *et al.*(34) | Colonic manometry (water perfused catheters); | Direct measurement of intraluminal colonic pressure; Distinguish between neuropathic and myopathic motility disorders; Help guide need for surgical management or constipation and if successful closure of ostomies; | Catheter placement under general anaesthesia; Inpatient admission; Radiation exposure; Specialised centres; Not able to distinguish patients in which Sacral Nerve Stimulation can be successful; |
| 2024 – Fu Y. *et al.*(42) | Colonic manometry; | Identification of neuromuscular dysfunction; Guidance for pathologies that might be responsive to surgical management; | Invasive; General anaesthetic; |
| 2024 – Rolleman N. *et al.*(89) | Surface electroenterography; | Non-invasive; Assessment of segmental colonic function; Measurement of gastrocolic reflex; Potential for ambulatory monitoring; | Movement artefact risk; Short recording duration; Technical challenges in the paediatric setting (body size); Not validated in paediatrics; |
| 2024 – van der Zande *et al.*(105) | 1) Colonic manometry;  2) Anorectal manometry; | 1) Direct colonic motor function assessment; Evaluation of physiologic responses;  2) Comprehensive assessment of anorectal physiology; | 1) Invasive; Bowel preparation;  2) Results may be influenced by general anaesthesia; Results may be influenced by patient anxiety; Limited assessment with patient under general anaesthesia; |
| 2025 – Chanpong A. *et al.*(5) | 1) Colonic Scintigraphy;  2) Colonic Manometry (water-perfused manometry catheter); | 1) Useful in predicting patients that are likely to have abnormalities in colonic manometry; | 1) Variation in protocols; Repeated imaging;  2) Variation in protocols; |
| 2025 – Dorfman L. *et al.*(37) | Colonic manometry; | Information about colonic contractility in different states; | Prolonged fasting phase has relatively low diagnostic yield; |
| 2025 – Dorfman L. *et al.*(36) | Colonic manometry; | Valuable diagnostic tool for refractory constipation, surgical planning, and Hirschsprung disease evaluation; | Invasive; Expensive and requires expertise; International protocol variability; |
| 2025 – Shan AYC. *et al.*(91) | 1) Colonic manometry;  2) Radiopaque Markers; | 1) Direct assessment of colonic motor activity;  2) Non-invasive; Simple; | 1) Invasive; Resource-intensive;  2) No information about colonic motor function; Influenced by multiple factors; Radiation; |
