# Supplementary material for: Thirty years of paediatric colonic motility assessment modalities: a systematic review
Source: Front Pediatr. 2026 Sep 18;14:1951800. doi: 10.3389/fped.2026.1951800 (PMC13630753; doi:10.3389/fped.2026.1951800)
Supplement: Supplementary file 3 [file Supplementaryfile3.docx]

**Appendix 3 - Table 5:** Risk of bias assessment using the Modified Newcastle-Ottawa Scale (NOS) for non-randomised trials (case studies, cohort studies and case-control studies). (*A study can be awarded a maximum of one star for each numbered item within the Selection and Outcome categories. A maximum of two stars can be given for Comparability)

| **Criteria** | **Study type** | **Selection** | | | | **Comparability** | **Outcome** | | | **Score** |
| --- | --- | --- | --- | --- | --- | --- | --- | --- | --- | --- |
|  |  | **Representativeness of the exposed cohort** | **Selection of the non-exposed cohort** | **Ascertainment of exposure** | **Demonstration that outcome of interest was not present at the start of the study** | **Comparability of cohorts on the basis of the design or analysis** | **Assessment of outcome** | **Was follow-up long enough for outcomes to occur** | **Adequacy of follow-up of cohorts** | ***=1, no*=0** |
| 1997 – Pigeon N. *et al.*(79) | Prospective case-control study | a)  * | a)  * | a)  * | a)  * | a) and b)  ** | a)  * | a)  * | a)  * | 9 |
| 1997 – Rintala RJ. *et al.*(84) | Prospective cohort study | a)  * | a)  * | a)  * | b) | b)  * | a)  * | a)  * | a)  * | 7 |
| 1998 – Hamid SA. *et al.*(124) | Prospective cohort study | a)  * | - | a)  * | a)  * | - | a)  * | b) | - | 4 |
| 1998 – Shankar KR. *et al.*(106) | Retrospective cohort | a)  * | - | a)  * | a)  * | - | a)  * | a)  * | a)  * | 6 |
| 1999 – Baillie CT. *et al.*(15) | Prospective cohort study | a)  * | a)  * | a)  * | b) | b)  * | b)  * | a)  * | a)  * | 7 |
| 1999 – Chung KK. *et al.*(23) | Prospective cohort study | b) | a)  * | a)  * | b) | - | b)  * | b) | d) | 3 |
| 1999 – Heikenen JB. *et al.*(51) | Prospective cohort study | a)  * | - | a)  * | b) | - | a)  * | a)  * | a)  * | 5 |
| 1999 – Ure BM. *et al.*(104) | Prospective cohort study | a)  * | a)  * | a)  * | b) | - | a)  * | a)  * | a)  * | 6 |
| 1999 – Wheatley JM. *et al.*(128) | Prospective cohort study | a)  * | - | a)  * | a)  * | - | a)  * | - | - | 4 |
| 2000 – Di Lorenzo CS. *et al.*(32) | Prospective cohort study | b) | b) | a)  * | b) | - | b)  * | a)  * | b)  * | 4 |
| 2000 – Lucanto C. *et al.*(68) | Retrospective case series | a)  * | b) | a)  * | b) | - | a)  * | a)  * | a)  * | 5 |
| 2000 – Miele ET. *et al.*(73) | Prospective cohort sutdy | a)  * | a)  * | a)  * | b) | b)  * | a)  * | a)  * | c) | 6 |
| 2001 – Treepongkaruna S. *et al.*(102) | Retrospective cohort study | a)  * | a)  * | a)  * | b) | - | a)  * | - | a)  * | 5 |
| 2001 – Guimaraes EV. *et al.*(49) | Prospective cross-sectional study | a)  * | b) | a)  * | a)  * | - | a)  * | - | a)  * | 5 |
| 2001 – Villarreal J. *et al*.(111) | Retrospective cohort study | a)  * | - | a)  * | a)  * | - | a)  * | a)  * | a)  * | 6 |
| 2002 – Gutierrez C. *et al.*(46) | Prospective case control study | a)  * | a)  * | a)  * | a)  * | b)  * | a)  * | a)  * | a)  * | 8 |
| 2002 – Shin YM. *et al.*(92) | Retrospective cohort study | a)  * | a)  * | a)  * | b) | - | a)  * | - | a)  * | 5 |
| 2003 – Pensabene L. *et al.*(78) | Retrospective cohort study | a)  * | - | a)  * | b) | - | a)  * | a)  * | c) | 4 |
| 2004 – Benninga MA. *et al.*(16) | Prospective cohort study | a)  * | a)  * | a)  * | b) | - | b)  * | b) | d) | 4 |
| 2004 – Chitkara DK. *et al.*(22) | Prospective cohort study | b) | a)  * | a)  * | b) | - | b)  * | b) | d) | 3 |
| 2004 – de Lorijn F. *et al.*(29) | Prospective cohort study | a)  * | a)  * | a)  * | a)  * | - | b)  * | a)  * | b)  * | 7 |
| 2004 – Langer J. *et al.*(63) | Retrospective case series | a)  * | - | a)  * | a)  * | - | a)  * | a)  * | a)  * | 6 |
| 2004 – Martin MJ. *et al.*(70) | Prospective cohort study | a)  * | - | a)  * | a)  * | - | a)  * | a)  * | a)  * | 6 |
| 2004 – Zaslavsky C. *et al.*(117) | Prospective cohort study | a)  * | a)  * | a)  * | a)  * | a)  * | a)  * | a)  * | a)  * | 8 |
| 2005 – Gertken JT. *et al.*(43) | Retrospective case series | a)  * | - | a)  * | - | - | a)  * | a)  * | a)  * | 5 |
| 2005 – Soares ACF. et al.(94) | Prospective cohort study | a)  * | a)  * | a)  * | a)  * | - | a)  * | a)  * | a)  * | 7 |
| 2005 – Stanton MP. *et al.*(98) | Prospective case-control study | a)  * | a)  * | a)  * | - | - | a)  * | - | a)  * | 5 |
| 2005 – Watanabe Y. *et al.*(114) | Prospective cohort study | a)  * | - | a)  * | - | - | a)  * | a)  * | a)  * | 5 |
| 2005 – Zhang SC. et al.(118) | Retrospective cohort study | a)  * | a)  * | a)  * | a)  * | b)  * | a)  * | a)  * | a)  * | 8 |
| 2006 – Zuccarello B. *et al.*(121) | Retrospective cohort study | a)  * | a)  * | a)  * | a)  * | b)  * | a)  * | a)  * | a)  * | 8 |
| 2007 – Bijos A. *et al.*(17) | Prospective cohort study | b) | a)  * | a)  * | b) | - | b)  * | b) | d) | 3 |
| 2007 – Kohno M. *et al.*(60) | Prospective cohort study | a)  * | - | a)  * | a)  * | - | a)  * | a)  * | a)  * | 6 |
| Zhang SC. *et al.*(119) | Retrospective cohort study | a)  * | a)  * | a)  * | a)  * | b)  * | a)  * | a)  * | a)  * | 8 |
| 2008 – Demirogullari B. *et al.(30)* | Prospective cohort study | b) | - | a)  * | a)  * | - | b)  * | a)  * | a)  * | 5 |
| 2008 – King SK. *et al.*(58) | Prospective case-control study | a)  * | a)  * | a)  * | a)  * | - | a)  * | a)  * | c) | 6 |
| 2008 – Venkatasubramani N. *et al.*(110) | Retrospective case series | a)  * | - | a)  * | a)  * | - | a)  * | a)  * | a)  * | 6 |
| 2008 – Yuan Z. *et al.*(116) | Prospective case-series study | a)  * | a)  * | a)  * | a)  * | b)  * | a)  * | a)  * | a)  * | 8 |
| 2009 – Clarke MCC. *et al.*(25) | Prospective cohort study | b) | - | a)  * | a)  * | - | b)  * | a)  * | a)  * | 5 |
| 2009 – Soares ACF. *et al.*(95) | Prospective cohort study | a)  * | - | a)  * | a)  * | - | a)  * | a)  * | a)  * | 6 |
| 2009 – Sutcliffe JR. *et al.*(100) | Retrospective cohort study | a)  * | - | a)  * | d) | - | a)  * | - | d) | 3 |
| 2010 – Dranove R*. et al.*(4) | Prospective cohort study | a)  * | - | a)  * | a)  * | - | a)  * | b) | d) | 4 |
| 2010 – Gomez R. *et al.*(45) | Prospective cohort study | a)  * | - | a)  * | a)  * | - | a)  * | b) | a)  * | 5 |
| 2010 – Koh HL. *et al.*(59) | Retrospective cohort study | a)  * | a)  * | a)  * | a)  * | - | a)  * | a)  * | - | 6 |
| 2010 – Liem OB. *et al.*(67) | Prospective cohort study | a)  * | a)  * | a)  * | b) | - | a)  * | - | a)  * | 5 |
| 2010 – Märzheuser S. *et al.*(71) | Prospective cohort study | a)  * | - | a)  * | a)  * | - | b)  * | a)  * | a)  * | 6 |
| 2011 – Grasshoff-Derr S. *et al.*(47) | Prospective cohort study | a)  * | - | a)  * | a)  * | - | a)  * | a)  * | a)  * | 6 |
| 2011 – Kaul A. *et al.*(56) | Retrospective cohort study | a)  * | a)  * | a)  * | b) | a) and b)  ** | a)  * | a)  * | a)  * | 8 |
| vd Baan-Slootweg OH. e*t at.*(105) | Prospective cohort study | a)  * | - | a)  * | - | - | a)  * | - | a)  * | 4 |
| 2012 – Clarke MCC. *et al.*(24) | Prospective cohort study | b) | - | a)  * | a)  * | - | b)  * | b) | a)  * | 4 |
| 2012 – Liem O. *et al.*(66) | Prospective cohort study | a)  * | - | a)  * | a)  * | a) and b)  ** | a)  * | - | - | 6 |
| 2012 – Rodriguez L. *et al.*(108) | Retrospective case series | a)  * | - | a)  * | - | - | a)  * | - | a)  * | 4 |
| 2012 – Tipnis NA. *et al*.(101) | Retrospective cohort study | a)  * | a)  * | a)  * | b) | a)  * | c) | a)  * | b)  * | 6 |
| 2012 – Yik YI. *et al.*(129) | Prospective cohort study | a)  * | - | a)  * | a)  * | - | a)  * | a)  * | a)  * | 6 |
| 2013 – Firestone Baum C. *et al.*(40) | Retrospective case series | a)  * | - | a)  * | b) | - | a)  * | - | d) | 3 |
| 2013 – Giorgio V. *et al.*(44) | Prospective cohort study | a)  * | a)  * | a)  * | a)  * | b)  * | a)  * | a)  * | a)  * | 8 |
| 2013 – Hedsund C. *et al.*(50) | Prospective cohort study | a)  * | c) | a)  * | a)  * | - | a)  * | a)  * | a)  * | 6 |
| 2013 – Keshtgar AS. *et al.*(57) | Prospective cohort study | a)  * | - | a)  * | b) | - | a)  * | a)  * | a)  * | 5 |
| 2013 – Marte A. *et al.*(125) | Prospective cohort study | a)  * | - | a)  * | a)  * | - | a)  * | a)  * | a)  * | 6 |
| 2013 – Mugie SM. *et al.*(74) | Retrospective cohort study | a)  * | - | a)  * | a)  * | - | a)  * | - | a)  * | 5 |
| 2013 – Rodriguez L. *et al.*(88) | Retrospective cohort study | a)  * | - | a)  * | a)  * | a)  * | a)  * | a)  * | c) | 6 |
| 2013 – Velde SV. *et al.*(107) | Prospective cohort study | a)  * | a)  * | a)  * | b) | - | a)  * | - | - | 4 |
| 2014 – El-Chammas KI. *et al.*(39) | Prospective cohort study | a)  * | - | a)  * | a)  * | - | a)  * | - | d) | 4 |
| 2014 – Kerur B. *et al.*(64) | Retrospective case series study | a)  * | b) | a)  * | b) | - | a) * | a)  * | a)  * | 5 |
| 2014 – Liem O. *et al.* | Prospective cohort study | a)  * | - | a)  * | a)  * | - | a)  * | - | - | 4 |
| 2014 – Zivkovic VD. *et al.*(120) | Prospective cohort study | a)  * | a)  * | a)  * | a)  * | b)  * | a)  * | a)  * | a)  * | 8 |
| 2015 – Carmo RLML. *et al.*(20) | Prospective cohort study | b) | a)  * | a)  * | a)  * | - | a)  * | a)  * | a)  * | 6 |
| 2015 – Jacobs C. *et al.*(54) | Retrospective Cohort study | a)  * | a)  * | a)  * | b) | a) and b)  ** | a)  * | a)  * | a)  * | 8 |
| 2015 – Queiroz Machado V. *et al.*(80) | Prospective cohort study | a)  * | - | a)  * | - | - | a)  * | b) | a)  * | 4 |
| 2015 – Quitadamo P. *et al.*(81) | Prospective cohort study | a)  * | - | a)  * | - | a) and b)  ** | a)  * | a)  * | a)  * | 7 |
| 2015 – Ridha Z. *et al.*(83) | Retrospective case series | a)  * | a)  * | a)  * | b) | - | a)  * | a)  * | c) | 5 |
| 2016 – Akotia DH. *et al.* (12) | Prospective cohort study | b) | a)  * | a)  * | a)  * | - | a)  * | b) | a)  * | 5 |
| 2016 – Borrelli OP. *et al.*(18) | Prospective cohort study | a)  * | - | a)  * | a)  * | - | b)  * | b) | a)  * | 5 |
| 2016 – Gomez-Suarez RA. *et al.*(48) | Retrospective cohort study | a)  * | - | a)  * | a)  * | - | a)  * | a)  * | a)  * | 6 |
| 2016 – Stathopoulos L. *et al.*(99) | Retrospective case series | a)  * | - | a)  * | b) | - | a)  * | a)  * | a)  * | 5 |
| 2016 – Wessel S. *et al.*(127) | Prospective cohort study | a)  * | b) | a)  * | a)  * | - | a)  * | - | - | 4 |
| 2017 - Arbizu RA. *et al.*(13) | Prospective cohort study | a)  * | - | a)  * | a)  * | - | b)  * | b) | a)  * | 5 |
| 2017 – Koppen IJN. *et al.*(60) | Retrospective cohort study | a)  * | a)  * | a)  * | a)  * | a)  * | a)  * | - | - | 6 |
| 2018 – Daeze C. *et al.*(122) | Prospective cohort study | a)  * | a)  * | a)  * | a)  * | b)  * | a)  * | a)  * | b) | 7 |
| 2018 – Koppen IJN. *et al.*(61) | Retrospective cohort study | a)  * | a)  * | a)  * | a)  * | a)  * | a)  * | - | - | 6 |
| 2018 – Sanghavi RM. *et al.*(90) | Retrospective case series | a)  * | - | a)  * | a)  * | - | a)  * | b) | a)  * | 5 |
| 2018 – Shava U. *et al.*(126) | Prospective case-control study | a)  * | a)  * | a)  * | a)  * | - | a)  * | a)  * | a)  * | 7 |
| 2019 – Sintusek P. *et al.*(93) | Retrospective case-control study | a)  * | a)  * | a)  * | b) | - | a)  * | - | a)  * | 5 |
| 2020 – Dranove J. *et al.*(33) | Retrospective cohort study | a)  * | a)  * | a)  * | a)  * | - | a)  * | b) | a)  * | 6 |
| 2020 – Heitmann PT. *et al.*(52) | Retrospective cohort study | a)  * | - | a)  * | b) | - | a)  * | a)  * | a)  * | 5 |
| 2020 - Rodriguez L. *et al.*(87) | Retrospective cohort study | a)  * | a)  * | a)  * | a)  * | - | b)  * | a)  * | b)  * | 7 |
| 2021 – Brinck CE. *et al.*(19) | Prospective cohort study | b) | - | a)  * | a)  * | - | b)  * | b) | b)  * | 4 |
| 2021 – De Sillos MD. *et al.*(31) | Prospective case series | a)  * | a)  * | a)  * | a)  * | - | b)  * | b) | a)  * | 6 |
| 2021 – Rodriguez L. *et al.*(86) | Prospective cohort study | a)  * | - | a)  * | a)  * | - | a)  * | a)  * | a)  * | 6 |
| 2021 – Vriesman MH. *et al.*(112) | Prospective cohort study | a)  * | - | a)  * | b) | - | a)  * | a)  * | c) | 4 |
| 2022 – Fritz T. *et al.*(41) | Retrospective case series | a)  * | - | a)  * | b) | - | a)  * | b) | d) | 3 |
| 2022 – Chen JJ. *et al.*(21) | Prospective cohort study | b) | a)  * | a)  * | a)  * | b) * | b)  * | a)  * | c) | 6 |
| 2022 – Infante JR. *et al.*(53) | Retrospective cohort study | a)  * | - | a)  * | b) | - | a)  * | a)  * | a)  * | 5 |
| 2022 – Rodriguez L. *et al.*(85) | Retrospective cohort study | a)  * | - | a)  * | a)  * | a)  * | a)  * | a)  * | c) | 6 |
| 2022 – Tsai A. *et al.*(103) | Retrospective case series | a)  * | - | a)  * | - | - | a)  * | . | a)  * | 4 |
| 2023 – Coe A. *et al.*(27) | Retrospective case-control study | b) | a)  * | a)  * | a)  * | a) and b)  ** | a)  * | b) | a)  * | 7 |
| 2023 – Colliard K. *et al.*(28) | Retrospective case series | b) | - | a)  * | a)  * | a) and b)  ** | b)  * | a)  * | a)  * | 7 |
| 2023 – Dorfman L. *et al.*(38) | Retrospective cohort study | a)  * | a)  * | a)  * | a)  * | - | a)  * | - | d) | 5 |
| 2023 – Jacobs SE. *et al.*(55) | Retrospective case series | a)  * | - | a)  * | b) | - | a)  * | a)  * | a)  * | 5 |
| 2023 – Mansi S. *et al.*(69) | Prospective cohort study | a)  * | b) | a)  * | a)  * | b)  * | a)  * | a)  * | a)  * | 7 |
| 2023 – Mehta V. *et al.*(72) | Prospective cohort study | a)  * | - | a)  * | a)  * | - | a)  * | - | a)  * | 5 |
| 2023 – Yuan T. *et al.*(115) | Prospective cohort study | a)  * | - | a)  * | a)  * | - | a)  * | a)  * | a)  * | 6 |
| 2024 – Dorfman L. *et al.*(35) | Retrospective cohort study | a)  * | - | a)  * | a)  * | - | b)  * | - | a)  * | 5 |
| 2024 – Dorfman L. *et al.*(34) | Retrospective cohort study | a)  * | a)  * | a)  * | a)  * | - | b)  * | a)  * | a)  * | 7 |
| 2024 – Fu Y. *et al.*(42) | Retrospective cohort study | a)  * | - | a)  * | b) | - | a)  * | a)  * | c) | 4 |
| 2024 – Rolleman N. *et al.*(89) | Prospective cohort study | a)  * | a)  * | a)  * | - | - | a)  * | - | a)  * | 5 |
| 2024 - Van der Zande *et al.*(105) | Retrospective case-control study | a)  * | a)  * | a)  * | a)  * | b)  * | a)  * | a)  * | a)  * | 8 |
| 2025 – Chanpong A. *et al.*(5) | Prospective cohort study | b) | - | a)  * | a)  * | - | b)  * | - | d) | 3 |
| 2025 – Dorfman L. *et al.*(37) | Retrospective cohort study | a)  * | - | a)  * | a)  * | - | a)  * | - | d) | 4 |
| 2025 – Dorfman L. *et al.*(36) | Cross sectional survey study | a)  * | - | b)  * | - | a)  * | b)  * | - | a)  * | 5 |
| 2025 – Shan AYC. *et al.*(91) | Retrospective cohort study | a)  * | - | a)  * | a)  * | - | a)  * | a)  * | c) | 5 |
