# Supplementary material for: Thirty years of paediatric colonic motility assessment modalities: a systematic review
Source: Front Pediatr. 2026 Sep 18;14:1951800. doi: 10.3389/fped.2026.1951800 (PMC13630753; doi:10.3389/fped.2026.1951800)
Supplement: Supplementary file 4 [file Supplementaryfile1.docx]

**Appendix 1: search strategy**

**COCHRANE**

**EMBASE**

**OVID MEDLINE**
